# Qualitative testing of potential modifications to the EQ-HWB-9 in the United Kingdom

**DOI:** 10.1186/s12955-026-02578-w

**Published:** 2026-07-14

**Authors:** Tessa Peasgood, Victoria Gale, Aisha Moolla, Jill Carlton, Clara Mukuria

**Affiliations:** 1 Sheffield Centre for Health and Related Research (SCHARR), School of Medicine and Population Health, University of Sheffield, Sheffield, UK; 2 School of Humanities and Social Sciences, Leeds Beckett University, Leeds, UK; 3 CM HE Consulting, Sheffield, UK

**Keywords:** EQ-HWB-9, Content validity, Modifications, Health and wellbeing, Quality of life, Cognitive interviews, Qualitative

## Abstract

**Background:**

The Experimental EQ Health and Wellbeing 9 (EQ-HWB-9) is a generic measure developed to assess the impact of health, social care and informal caregiving on quality of life. Several modifications have been proposed based on emerging evidence. This study aimed to assess the content validity of these modifications and user preferences.

**Method:**

Individuals with a long-standing physical or mental health condition or disability were invited from an online panel ensuring gender balance and age coverage. Two interviewers conducted semi-structured cognitive interviews to assess comprehensibility, relevance, and comprehensiveness of the modified experimental EQ-HWB-9 v1.2. Participants’ views on differences between the modified and original experimental versions, as well as their preferences, were explored. Interviews were conducted via videoconferencing, recorded and transcribed verbatim. Framework analysis was used to code interviews, with themes drawn from the literature and data.

**Results:**

Twenty-six interviews were completed. Half the participants were female; participant age ranged from 24 to 68 years. Six of the nine items (‘activities’, ‘exhausted’, ‘lonely’, ‘difficulty concentrating/thinking clearly’, ‘anxious’, ‘sad/depressed’) were understood as intended by all participants. Some comprehension issues arose with the ‘mobility’ and ‘pain_severity’ items. Participants generally preferred the modified version, though a clear majority was not always observed. Justifications focused on item appropriateness, readability and distress.

**Discussion and conclusion:**

As a generic measure, EQ-HWB-9 v1.2 performed well for comprehension, relevance and comprehensiveness. Modifications were well accepted and, except for the use of item stem, preferred over the original version. These findings will be combined with other international evidence to support finalising the instrument.

**Supplementary Information:**

The online version contains supplementary material available at https://doi.org/10.1186/s12955-026-02578-w.

## Introduction

The EQ-Health and Wellbeing (EQ-HWB) is a generic measure designed to assess the impact of health, social care and informal caregiving on quality of life [1]. The 25 item EQ-HWB and its 9-item short form EQ-HWB-9 were developed through rigorous testing of face validity and psychometric properties of the item pool in six countries, involving patients, carers and social care users [2, 3]. Following the initial development the measures were designated ‘Experimental’ for further validation as stand-alone instruments in their formatted layout and possible refinement. Evidence gathered to date provided an opportunity to refine the measures while they remained experimental. Proposed modifications to the EQ-HWB-9 v1.1 included revising two of the response options, modifying the wording on three items and modifying the layout and order for two items (see Supplement 1 for details). These modifications were proposed by the EuroQol’s EQ-HWB Working Group following reflection on the findings of validity testing across multiple countries and groups [4–13], pilot valuation studies [14, 15] and translation work undertaken by EuroQol’s Version Management Committee (VMC).

Content validity is arguably the most important measurement property of a patient-reported outcome measure [16]. It is therefore important to assess whether these modifications perform as intended, enhancing usability without introducing new issues and confirm that the items are comprehensible in their final form [17, 18]. As the source language for other translations ensuring comprehensibility in English is particularly important. Cognitive interviews are particularly suited to this task, providing in-depth insight into understanding, relevance, and comprehensiveness [19, 20]. This study evaluates the content validity of the EQ-HWB-9 experimental modified version (EQ-HWB-9 v1.2) in UK sample with diverse health conditions.

## Methods

A qualitative cognitive interview study was conducted to evaluate the content validity of the modified EQ-HWB-9. Primary objectives were to assess:

1. Comprehensibility i.e., whether instructions and items in the EQ-HWB-9 v1.2 were understood and interpreted as intended.
2. Perceived differences and preferences between alternative item wording i.e., participants’ views on modified vs. original versions of the experimental EQ-HWB-9.

Secondary objectives were to assess:

3. Relevance i.e., whether items reflected the impact of health conditions or disabilities on quality of life.
4. Comprehensiveness i.e., whether any important aspects were missing.

To achieve the objectives, the EQ-HWB-9 v1.2 was evaluated through semi-structured one-to-one qualitative interviews. Objectives 3 and 4 were secondary because, as a generic instrument, some items may not be relevant to every participant. An additional card ranking task for the modified frequency response options was included to provide additional verification that they were interpreted in the intended hierarchical order, supplementing the evidence from verbal probing and providing relevant context for future valuation work. The modified difficulty response option (i.e. switching ‘some’ to ‘moderate’) was not included as this change aligns the EQ-HWB-9 to the EQ-5D-5L response options, which have demonstrated good performance in measurement and valuation.

### Participants

Adults (18 + years) residing in the UK were recruited via Prolific (prolific.com), an online research platform that maintains a panel of pre-screened participants and is widely used in academic research. Based on pre-screening data, participants with a longstanding physical or mental health condition or disability were invited, ensuring gender balance and age diversity. Invitees could also self-identify as carers and/or social care users, however, during the interview they were asked to reflect on how the questionnaire related to their experiences in terms of their health condition. Participants were reimbursed £15 through Prolific. Ethical approval was granted by the University of Sheffield, reference 064729.

The sample size was guided by the need to achieve diversity across health conditions, age groups (18 to 44, 45 to 64, 65 or over) and gender. Whilst COSMIN recommends a minimum of seven participants for content validity studies [16], cognitive interviewing practice suggests sample size should be determined by the diversity of the target population rather than saturation [20]. A sample of approximately 25 participants was therefore considered sufficient.

### Interview process

Before the interview, participants were given information about the study and provided consent via an online survey. An interview topic guide (see Supplement 2) was developed following the COSMIN framework for assessing content validity [16] and drew on prior work with the EQ-HWB and other EuroQoL instruments. Interviews were conducted online using Google Meet and video recorded and lasted approximately 50 min. Cognitive interviewing has been successfully conducted remotely [21].

Each interview began with a warm-up task to familiarize participants with the study’s focus on the impacts of health upon quality of life. Participants then completed an online card ranking exercise to explore how they ordered the modified frequency response options (“none of the time,” “a little of the time,” “sometimes,” “often,” “most or all of the time”). Next, they reviewed each item in the EQ-HWB-9 v1.2 responding to verbal probing [22] and sharing their understanding. Probing explored whether participants’ interpretations aligned with the intended meaning. Participants were also asked whether each item was relevant to their own quality of life. After reviewing the full EQ-HWB-9 v1.2 and considering its comprehensiveness, participants compared each modification to the original version. They reflected on whether changes affected meaning or how they would respond, and stated any preferences between versions, explaining their reasons. The recall period was not explored directly during the interviews.

During the interview, participants completed a brief demographic questionnaire displayed on-screen via Qualtrics and administered verbally by the interviewer. The questionnaire covered participants’ health history (duration of health problems, social care use, and informal caregiving), general health status (excellent to poor), long-term health impact, life satisfaction (0–10 scale), education level, and employment status.

Two researchers with health sciences backgrounds conducted the interviews (VG and AM) after receiving in-person training by members of the research team that included multiple pilot sessions to refine presentation of study elements and ensure alignment with objectives with input from the research team. Data from these pilot interviews were excluded from analysis. The team held regular discussions during data collection to review progress and resolve any emergent issues. The protocol was amended after the first four interviews; due to time pressures, completion of the EQ-HWB-9 was removed from the interview.

### Analysis

The interviews were transcribed intelligent verbatim (whereby filler words and repetitions are omitted while preserving the meaning of speech) using automatic software and checked for accuracy by the interviewers. Framework analysis was used which involved familiarisation with the interview data, refining the theoretical framework, coding transcripts, charting the individual data onto Excel with rows as individuals, columns as themes, and cells containing summaries and illustrative quotes, and finally, the mapping stage for synthesis and interpretation [23]. A pre-defined framework in Excel based on content validity dimensions of comprehensibility, relevance and comprehensiveness was used, with themes related to preferences developed inductively. In the first stage, each item was coded for (i) Understanding (Yes, No, Partial, with potentially multiple codes for same individual), (ii) Relevance (Yes, No, Uncertain), (iii) perceived difference between the modified and original items or response options, (iv) preferences between modified and original items (preference for modified/original/no preference). At initial coding, multiple codes for Understanding and Relevance could be used for the same individual; these cases and those with Partial and Uncertain codes were flagged as cases requiring further discussion. An internal EuroQol intended meaning document for each item was used to check for comprehensibility and support coding alignment.

The two interviewers independently coded the same two interviews, then met with the research team to discuss any disagreements and refine the coding framework. This process was repeated with two additional interviews to ensure alignment between coders and finalise the framework. Interviewers then coded their remaining own interviews. A further team meeting was held after coding to discuss and reflect on the analysis. In the second stage, coding was verified by the instrument developers (TM and CM) who jointly confirmed the coding and reassessed partial or multiple codes to a single code for each respondent for each question. Any disagreements were resolved through discussion and returning to the full transcripts. Codes which flagged concerns with understanding and relevance were additionally coded to categorise the source of misunderstanding or lack of relevance based on other content validity studies [4, 24] (see Supplement 3). Themes related to perceived differences and preferences between modifications and the original version were developed iteratively from the data. Where it was difficult to ascertain understanding, relevance or preferences from what participants said, this was coded as ‘unclear’. Data synthesis collated all responses, with particular attention given to misunderstandings of items and divergent viewpoints.

## Results

### Participants

Twenty-six participants were interviewed between January and April 2025. Half were female, with ages ranging from 24 to 68 years. The majority were white (88%), had at least bachelor’s degree level education (65%) and half were employed. Participants reported ten different health conditions (see Table S1 for details); six also identified as informal carers, and two reported using social care services. Each interview was identified using the interviewer initials (VG/AM), interview number, sex (F/M), age group (18–44, 45–64, 65+), and whether they are also a carer (C) e.g. VG04 M18-44_C.

### Card ranking exercise

Most participants (22 of 26) ranked the cards in the expected order. One viewed “a little of the time” and “sometimes” as equivalent, another ranked “sometimes” below “a little of the time”, one placed “most or all of the time” below “often,” and one did not understand the task.

### Content validity of the modified EQ-HWB-9

#### Comprehensibility

**Fig. 1:**
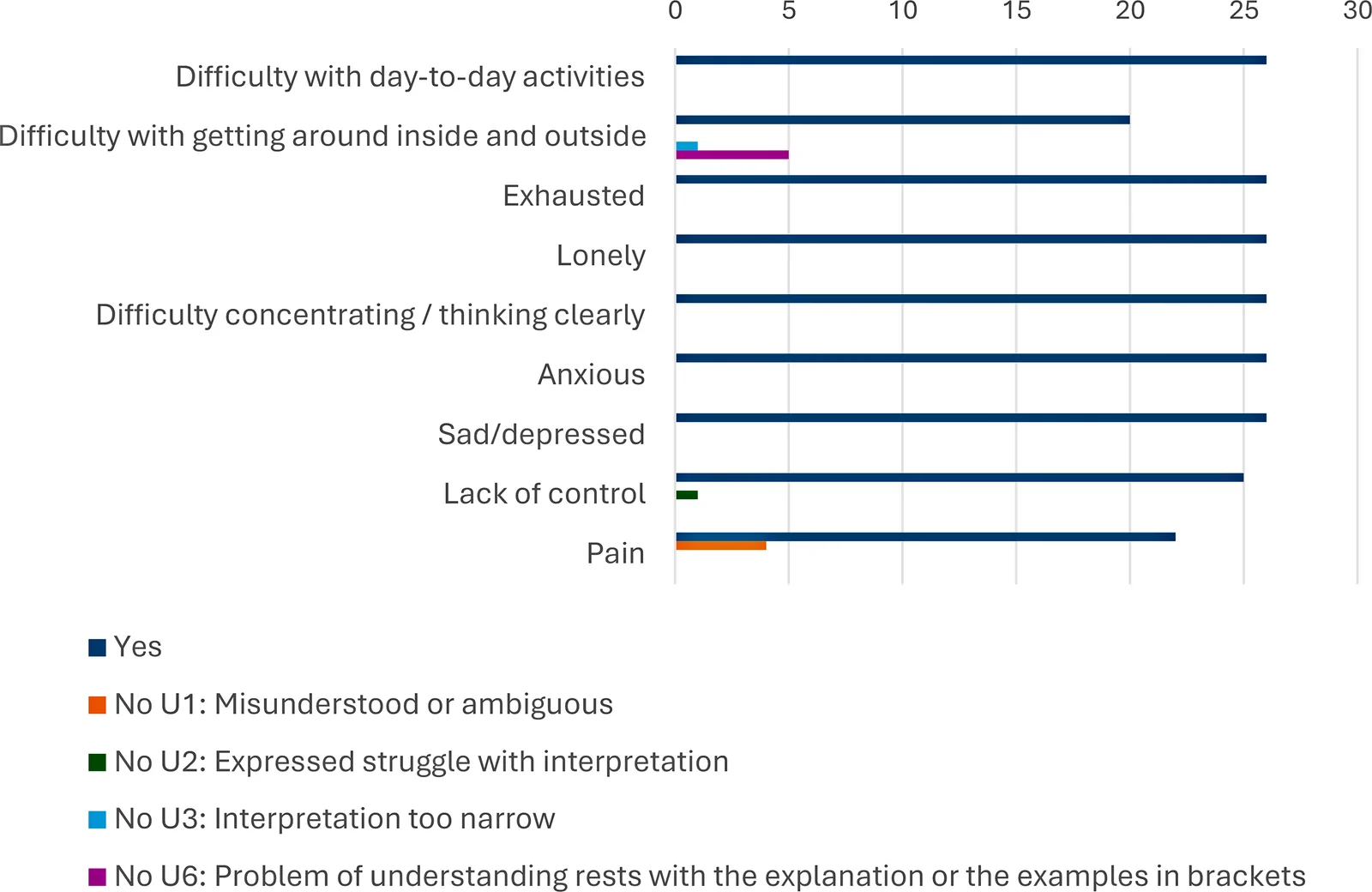
Number of interviews demonstrating understanding as intended for each item

Six items (‘activities’, ‘exhausted’, ‘lonely’, ‘thinking’, ‘anxious’, ‘sad_depressed’) were understood as intended by all participants. Illustrative quotes are shown in Supplementary Table S2, with an overview by item shown in Fig. 1.

The ‘mobility’ item posed challenges for six participants. One participant interpreted it as referring only to severe mobility limitations, stating, “…*so obviously this is I guess a slightly more extreme version of the previous question because it’s seeing if your mobility is restricted in any major way*” (AM06 M45-64). The other five participants understood the item as asking about the use of aids, such as a stick or wheelchair, e.g., “*It’s asking if I can walk without a stick or wheelchair*” (VG11 M65+).

One participant expressed difficulty interpreting the ‘control’ item, questioning whether self-perception of control aligned with reality and whether commitment to a job reduced control e.g. “*… for example okay I work remotely two days a week but for whatever reason if I need sometimes my work might say okay I have to be in office on this day because there is something happening […] In such a situation it’s objectively I’m being told do this right I’m basically given an order and so I sort of don’t have control over my life… So this question makes me wonder which of these sort of two possibilities it’s aiming at and I’m not actually sure.”* (VG03 M18-44). They did not relate lack of control to day-to-day independence which is a key part of the intended meaning.

The ‘pain’ item was slightly misinterpreted by four participants who questioned whether the question asked about frequency or severity e.g., “*I don’t know if we’re sort of trying to estimate like what proportion of the time have I been in pain or how severe has the pain been*” (VG02 F18-44). The ‘severity’ response options were also interpreted by some in terms of frequency, *“… being in severe pain*,* which for me means it’s there a lot of the time. Moderate pain is for me sort of transient”* (VG13 F65+). Others noted that pain requires two constructs, “*they talk about mild*,* moderate*,* very severe but there are two factors here. One the intensity and two is the frequency”* (VG03 M18-44).

#### Relevance

**Fig. 2:**
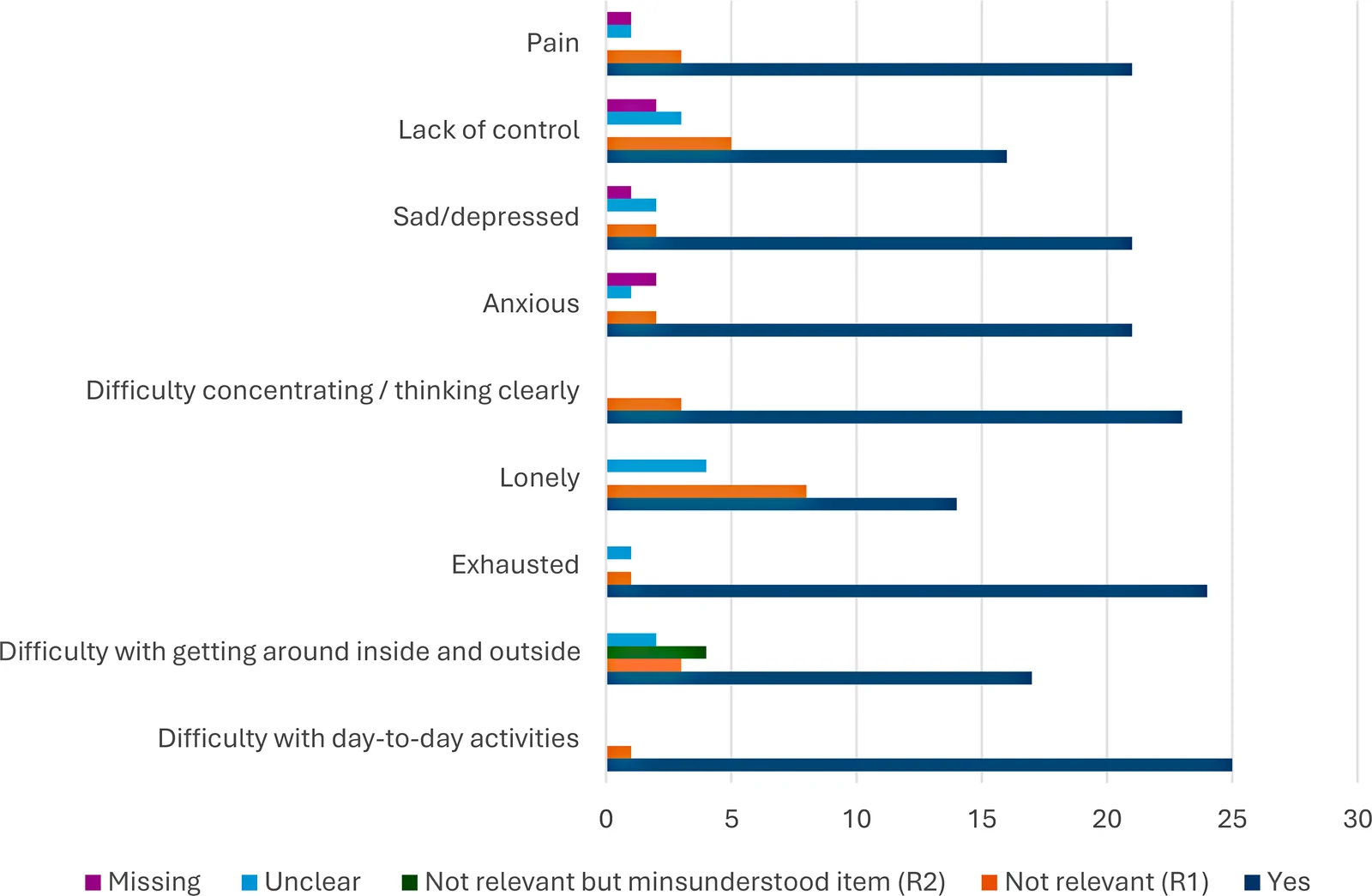
Number of interviews demonstrating relevance for each item

All the items had at least one or more participant who considered the question not relevant to their health condition, with loneliness (8/26) and control (5/26) having the highest rates of non-relevance. The ‘mobility’ item had four participants who said it was not relevant due to misunderstanding rather than genuine irrelevance e.g. “*I don’t use a walking stick. I’m not in a wheelchair. So for me it’s not particularly relevant but I understand in that case I’d say no difficulty.” (AM08 M65+)*. Supporting quotes are shown in Supplementary Table S3, with an overview by item shown in Fig. 2.

#### Comprehensiveness

Comprehensiveness was discussed in 11 interviews; the others did not have enough time (see Table S4 for quotes). Of these, four identified no missing items, seven identified missing information including spiritual health (*n* = 1/11), access to health care (*n* = 2/11) and access to support (*n* = 2/11). One participant noted the importance of contextualisation of their problems while another noted that a tick box questionnaire cannot fully capture an individualised judgement of quality of life.

Eight of the ten participants who discussed potentially redundant items did not consider any items to be redundant while two considered merging items, one suggesting merging the anxiety and sad/depressed items the other merging the day-to-day activities and getting around items (see Table S5).

### Preferences for potential modifications of the EQ-HWB-9

The reasons supporting preferences were grouped into three main themes: ‘Appropriateness’, ‘Readability’ and ‘Distressing or unpleasantness’. ‘Appropriateness’ covered views that the item, response option or layout fit the context better (e.g. a medical context), or had a more relevant scope or improved flow in relation to other items or was more accurate. ‘Readability’ referred to issues related to simplicity and the ease of reading. ‘Distressing or unpleasantness’ related to wording that caused negative emotions. Some participants could not provide a clear rationale for their preferences and these were grouped together, e.g. where respondents said their preferences were driven by intuition, or by the order in which they were seen etc.

Detailed summaries and supportive quotes can be found in Supplementary Materials S6 to S13b.

#### Modification of layout

One of the modifications being tested was the use of an item stem for the ‘mobility’ and ‘activities’ items compared with using two separate full questions. Participants expressing a preference had mixed views with slightly more (n = 13/24) preferring full sentences than the abbreviated stem (n = 11/24). Full sentences were preferred because it made instructions clear, *“I think having it there makes it less likely that people are going to miss it.” (VG02 F18-44)* and for ease of reading (Table S6). The shorter stem was preferred for ease of reading because it was faster or cleaner/more appealing “*I think that is easier*,* clearer to read really than reading it again” (AM11 F45-64)*.

Regarding item order, of those participants expressing a preference more preferred to see the ‘activities’ item first (n = 11/18) because this was considered more appropriate due to the nature of the items “*I think with the day-to-day activities it’s more general so it makes you sort of think about your day and then the second one is more specific …” (AM07 F18-44) (*Table S7). Conversely, seven participants preferred the ‘mobility’ item first due to the nature of the item or because it flowed better *“I feel like if you ask about your mobility first*,* it would make more sense because that impacts your day-to-day activity” (VG06 F18-44).* Three participants who preferred the ‘mobility’ item first noted they would change how they answered the activities by making them “*think in a more rounded way*” (AM10 F45-64) or give them a “*gentle prompt*” (AM02 M45-64) to consider activities outside of the home.

#### Item modifications

For item wording modifications respondents were first asked if they perceived the two options presented as different and if so why, then were asked which they preferred and why.

Most participants (*n* = 21/24) thought the meaning of the ‘mobility’ item changed when comparing ‘getting around inside *or* outside’ to ‘inside *and* outside’. Participants thought using ‘and’ implied problems must occur in both domains, whereas ‘or’ did not (Table S8a). Three thought they meant the same, and the rest were missing or unclear.

Fourteen participants (out of 19 expressing a clear preference) preferred the use of ‘or’ as they considered it more appropriate (Table S8b). This was because it allowed differences in settings e.g. *“If you’re only going to ask one question*,* I prefer the first one because you’re basically saying*,* are you mobile? And if you can only do inside*,* that’s mobile…” (AM08 M65+)*, and was relevant for those who have not been outside in the last seven days “*If I’m housebound and the question says inside and outside*,* I have a moment of thought of like but I don’t go outside*,* so how does this apply to me?”* (VG02 F18-44). However, for some (n = 5/19) the ‘or’ framing risks respondents focusing on one setting hence limits the comprehensiveness of the item and they preferred ‘and’. Of the remaining, two preferred two separate questions, one had no preference, one was coded as unclear, two had preferences that were based on misunderstanding, and one noted their preference would depend upon the item intention.

When considering the ‘concentrating/thinking clearly’ item, participants were split on whether the switch of the word ‘trouble’ to ‘difficulty’ changed the meaning of the item (Table S9a). Eight participants perceived that the use of the term ‘difficulty’ seemed more severe.

Most participants preferred the term ‘difficulty’ (*n* = 17), as they judged this to be more appropriate and less ambiguous e.g., “*Difficulty is how much problem you have … while trouble is a bit more ambiguous*” (VG04 M18-44_C), with one noting that ‘trouble’ seemed patronising (Table S9b). Four participants preferred the term trouble, for being a simpler way of asking the question and having a perceived broader coverage.

All the participants considered the meaning of the modification ‘lacked control’ to be different to the original ‘no control’, which was interpreted as more absolute (Table S10a).

Most participants preferred the ‘lacked control’ (*n* = 18/20 expressing a preference) with the main reason being that this was more appropriate due to the scope covered e.g., “*because I might feel that I’ve not got control in certain areas over my day-to-day life*,* but I have in others” (VG13 F65+*) (Table S10b) and less distressing. Two participants preferred the original (no control) because it was more appropriate as it related to external circumstances or signalled a problem. Six participants had no clear preference, five of whom considered ‘lack of control’ a better option if the question was aiming to pick up a feeling of slight loss or limitation in control.

The modified ‘control’ item also had changes in the explanation in the brackets, but this comparison was not covered by most participants (*n* = 22/26) due to interview time constraints (Table S11a). The four who provided preferences had split views, with two viewing the modified bracket wording as easier to read, and two viewing the original wording as clearer and more appropriate for explaining the meaning of lacking control for people receiving in-home care (Table S11b).

#### Response option modifications

Ten participants (out of 24 giving a response) considered ‘A little of the time’ to be different to ‘Only occasionally’ as a level two frequency response option, while 14 thought they were the same (Table S12a). ‘Only occasionally’ was seen as reporting something occurring less often by some, more sporadic, and both more and less specific.

Most participants preferred ‘a little of the time’ (n = 16/23 giving a justified preference) as it fit with the other frequency response options e.g. “*I think that that fits the scale to me better than occasionally.” (AM02 M45-64)*, was less ambiguous e.g. *“It’s less ambiguous” (AM10 F45-64)* and because *“There’s a difference between little and sometimes. There isn’t really a difference between occasionally and sometimes.” (VG03 M18-44)* (Table S12b). It was also viewed as more appropriate when thinking about the last seven days unlike only occasionally which was viewed as representing rare events. Two participants reported that the ‘only’ in only occasionally seemed to be slightly judgemental and minimising their problems. Participants held differing views on which option was clearest and least likely to cause ambiguity.

Most participants (*n* = 19/26) considered ‘moderate’ difficulty to be different to ‘some’ difficulty in the difficulty response options (Table S13a). Most of the differences were based on severity with ‘moderate’ judged as more severe than ‘some’.

Fifteen participants out of 22 who gave a justified preference preferred ‘moderate’ difficulty as they either considered it to be more appropriate for the difficulty scale and the previous level (slight difficulty) e.g. *“I prefer moderate actually because slight difficulties is some difficulty*,* isn’t it?” (AM01 M65+)* or because they considered it easier to understand than ‘some’, or more appropriate due to better reflecting ongoing difficulties (Table S13b).

However, similar reasons were given by those who preferred ‘some’ (*n* = 7/22) viewing it as easier to understand and more specific, “*…some people might find it difficult to categorize what moderation or moderate difficulty meant” (AM05 F45-64_C)*.

## Discussion

Overall, participants tended to prefer the modified versions, though preferences were not always clear-cut. For the first two items, the full original wording was slightly favoured over the stem format, though participants preferred the modified item order. Six items (‘activities,’ ‘exhausted,’ ‘lonely,’ ‘thinking,’ ‘anxious,’ ‘sad_depressed’) were understood as intended by all participants; the revised ‘thinking’ item was preferred, with ‘difficulty’ judged less ambiguous than ‘trouble’.

Three items (‘mobility,’ ‘control,’ and ‘pain_severity’) presented some challenges in interpretation. The modification to the ‘mobility’ item (inside ‘or’ vs. ‘and’ outside) was not problematic, and there was a slight overall preference for the revised version. However, some participants interpreted the question as focusing on the use of aids rather than mobility itself. The item was intended to capture mobility while accounting for the use of aids, given their relevance in a social care context, but the wording led to some confusion. The challenge of developing a mobility item that includes text to describe how it should be answered by those who use a mobility aid without risking misunderstanding for those who do not use mobility aids has been found with other instruments such as the Quality of Life – Aged Care Consumers (QOL-ACC) measure [25, 26]. Our findings suggest further testing of this item may be required.

There was a clear preference for the modified ‘control’ item over the original, due to the wording ‘lack of control’ being favoured over ‘no control.’ Most participants did not comment on the bracketed wording, and among those who did, preferences were mixed. Since only one participant struggled with the modified item, it can be inferred that the bracketed words functioned as intended.

The ‘pain_severity’ item was generally well understood; however, some participants questioned whether it referred to pain frequency or severity. This uncertainty may have been influenced by the use of the phrase ‘how much,’ or by the limitation of assessing pain with a single item when both frequency and severity are important to people. The phrase ‘how much’ was an amendment from the original EQ-HWB designed to improve the formatting of the pain item, however, this finding suggests that the inclusion of this term adds unnecessary confusion and should be reconsidered.

When considering response options, participants generally preferred the alternative difficulty option ‘moderate difficulty’ over ‘some difficulty’. Most also felt ‘a little of the time’ was more appropriate than ‘only occasionally’. In the card ranking exercise, most participants who completed the task (*n* = 22/25) were able to distinguish between the modified frequency response options. However, two participants viewed ‘a little of the time’ as equivalent or more frequent than ‘sometimes’, the next response level. This may relate to the ambiguity of ‘sometimes’ which can be interpreted as ‘a little’ or ‘a lot’. One participant also transposed the worst two response options. Whilst both the card sort and qualitative findings support the change to the modified response options, they also point to need to address the fact that a minority may still interpret response options differently when seen out of context and this should be taken into consideration in any valuation exercise.

Most items were perceived as relevant across this diverse group. Relevance was slightly lower for loneliness and control, however, as a generic instrument this is expected, as not all aspects of quality of life are affected by every health condition.

### Strengths and limitations

Modifications to the EQ-HWB-9 were tested rigorously in a diverse UK sample spanning a range of ages and health conditions. The sample size (*n* = 26), while large for qualitative research, did not cover all health conditions, minority ethnic groups, or all populations for whom the instrument is intended. Saturation was not formally assessed during data collection; however, as this study forms part of a larger international content validity programme, patterns identified here will be considered alongside findings from other national studies to form final instrument decisions. The use of verbal probing in the cognitive interviews, enabled in depth understanding of how participants interpret each item. However, this approach may have encouraged participants to ‘overthink’ their interpretation in ways that may not reflect how they may complete the instrument in practice [27], and may have heightened order effects whereby detailed probing on earlier items could prime or influence how participants interpreted and responded to subsequent items. It will be important to consider whether comprehension issues identified here in the mobility and pain items are replicated in other studies.

The sample was recruited via an online research panel and skewed towards higher educational attainment limiting generalisability. The broader international validation programme will help address these gaps.

Although interviews were conducted by researchers independent of the instrument development team, developer involvement in the coding and interpretation of findings is a recognised source of potential bias in content validity studies [16, 20]. The team were conscious of their own potential biases throughout. Further reflexive considerations across the research team are discussed in Supplement 4.

## Conclusion

As a generic measure of health-related, carer-related and social-care related quality of life, the EQ-HWB-9 v1.2 performed well in terms of understanding, comprehensiveness, and relevance. Overall, the findings suggest that the modified EQ-HWB-9 was well understood, with participants generally preferring the revised version over the original experimental version in all but one instance (use of the item stem). Some further refinement may be needed, particularly to clarify the ‘mobility’ item in relation to the use of aids. These findings will be combined with international evidence to support the finalisation of the instrument including further psychometric evaluation of the modified version.

## Supplementary Information

Below is the link to the electronic supplementary material.

Supplementary Material 1

## Data Availability

The Topic Guide for the interviews is shared in the Supplementary Appendix. Individual participant data (virbatim quotes, after deidentification) that underlie the results reported in this article, after are reported in the manuscript, tables and appendicies.
