# Supplementary material for: Qualitative testing of potential modifications to the EQ-HWB-9 in the United Kingdom
Source: Health Qual Life Outcomes. 2026 Jul 14;24:130. doi: 10.1186/s12955-026-02578-w (PMC13647529; doi:10.1186/s12955-026-02578-w)
Supplement: Supplementary file 1 [file 12955_2026_2578_MOESM1_ESM.docx]

**Supplementary materials:** **Qualitative testing of potential modifications to the EQ-HWB-9 in the UK**

Contents

[Supplement 1: EQ-HWB-9 Proposed Modifications for Testing	3](#_Toc221811914)

[Supplement 2: Topic Guide	5](#_Toc221811915)

[Supplement 3: Coding framework	13](#_Toc221811916)

[Supplement 4: Reflective considerations	14](#_Toc221811917)

[Supplement 5: Tables	15](#_Toc221811918)

[Table S1: Description of Participants (n=26)	15](#_Toc221811919)

[Table S2 Quotes for item understanding in EQ-HWB-9	16](#_Toc221811920)

[Table S3 Quotes for item relevance for EQ-HWB-9	21](#_Toc221811921)

[Table S4 Quotes for comprehensiveness (missing items) of EQ-HWB-9	28](#_Toc221811922)

[Table S5 Quotes for comprehensiveness (redundant) of EQ-HWB-9	29](#_Toc221811923)

[Table S6: Themes and quotes supporting preferences for modification of layout – use of stem for first two items vs repeated text	30](#_Toc221811924)

[Table S7: Themes and quotes supporting preferences for modification of layout order of items: activities first vs mobility first	32](#_Toc221811925)

[Table S8a: Summary of the themes for perceived difference between version of the item Get around: and vs or	36](#_Toc221811926)

[Table S8b: Themes and quotes supporting preferences for modification of the item Get around: and vs or	36](#_Toc221811927)

[Table S9a: Summary of themes for perceived differences for versions of the item Concentrating/Thinking clearly: Difficulty vs Trouble	40](#_Toc221811928)

[Table S9b: Themes and quotes supporting preferences for modification of the item Concentrating/Thinking clearly: Difficulty vs Trouble	40](#_Toc221811929)

[Table S10a: Summary of themes for perceived differences for item control: Lacked control vs no control	44](#_Toc221811930)

[Table S10b Themes and quotes supporting preferences for modification of item control: Lacked control vs no control	44](#_Toc221811931)

[Table S11a: Summary of themes for perceived differences for modification explanation in brackets in control item modified version vs original version	49](#_Toc221811932)

[Table S11b: Themes and quotes supporting preferences for modification of item control: explanation in brackets modified version vs original version	49](#_Toc221811933)

[Table S12a: Summary of perceived differences between response options ‘a little of the time’ vs ‘only occasionally	50](#_Toc221811934)

[Table S12b: Themes and quotes supporting preferences for modification for response options ‘a little of the time’ vs ‘only occasionally	50](#_Toc221811935)

[Table S13a: Summary of perceived differences between response options ‘moderate’ vs ‘some’	53](#_Toc221811936)

[Table S13b: Summary of themes and quotes on preferences for response options ‘moderate’ vs ‘some’	53](#_Toc221811937)

## Supplement 1: EQ-HWB-9 Proposed Modifications for Testing

| **Instructions for completion** | **Amend (Y/N)** | **Proposed modifications** |
| --- | --- | --- |
| These questions are about the last 7 days. | Y | Propose change to ‘These questions are about your health and wellbeing in the last 7 days.’ to inform respondents about the scope of the forthcoming questions. |
| Please answer all questions. There are no wrong or right answers. | Y | Propose removal of the statement to simplify. |
| Please select one response for each question. | Y | Propose change from **‘select’ to ‘choose’** to align self-report version with interviewer administered version. |
| **EQ-HWB-9 items (Response level D/F/S)**  SAMPLE | **Amend (Y/N)** | **Proposed modifications** |
| *In the last 7 days:* |  |  |
| How much difficulty did you have getting around inside and outside? (using e.g. a walking stick or wheelchair if you normally use them) (D) | Y | 1. Propose **change of order** with the question about ‘day-to-day activities’ appearing first to improve acceptability for carers.  2. Propose **removing the stem** (*how much difficulty did you have:”)* as a common statement above the item to shorten and simplify the item.  3. Propose change from ‘**inside and outside’ to ‘inside or outside’** to allow reporting of problems in either domain. |
| How much difficulty did you have doing day-to-day activities? (e.g. working, shopping, housework) (D) | Y | 1. Propose **change of order** as noted above.  2. Propose **removing the stem** as noted above. |
| *In the last 7 days, did you:* |  |  |
| feel exhausted? (F) | N | No change proposed other than to the response options. |
| feel lonely? (F) | N | No change proposed other than to the response options. |
| have trouble concentrating or thinking clearly? (F) | Y | Propose change of wording from **‘trouble’ to ‘difficulty’** to improve translatability. |
| feel anxious? (F) | N | No change proposed other than to the response options. |
| feel sad or depressed? (F)  SAMPLE | N | No change proposed other than to the response options. |
| feel you had no control over your day-to-day life? (e.g. had no choice to do things or have things done for you as you liked and when you wanted) (F) | Y | Propose change of wording of the item and the explanation in brackets to ‘feel you **lacked control** over your day-to-day life? (**e****.g. you had no choice over what you did or how things were done for you)’** to reduce ambiguity. |
| Please select **one** response to describe how much physical pain you had in the last 7 days. Did you have*:*  no / mild / moderate / severe / very severe physical pain? (S) | N | No change proposed other than changing **‘select’ to ‘choose’.** |
| **Response levels** | **Amend (Y/N)** | **Proposed modifications** |
| Difficulty (D): no / slight / some / a lot of difficulty / unable | Y | Change ‘**some’ to ‘moderate’** to reduce ambiguity. |
| Frequency (F): none of the time / only occasionally / sometimes / often / most or all of the time | Y | Change ‘**only occasionally’ to ‘a little of the time"** to reduce ambiguity. |
| Severity (S): no / mild / moderate / severe / very severe | N | No change proposed. |

***© EuroQol Research Foundation. EQ-HWB^TM^ is a trademark of the EuroQol Research Foundation. This is a modified EQ-HWB. Reproduced by permission of EuroQol Research Foundation. Reproduction of this version is not allowed. For reproduction, use or modification of the EQ-HWB (any version), please register your study by using the online EQ registration page:*** [***www.euroqol.org***](http://www.euroqol.org)

## Supplement 2: Topic Guide

**Aims and Objectives**

- To provide evidence to support decision-making on the final content for the EQ-HWB-S
- To test wording of modified items, including the examples provided in brackets and the response options & consider any possible alternative changes to examples, or alternatives to the text
- To test understanding (i.e., comprehensibility) of the modified items
- To test relevance of the modified items
  - Across different types of responders (patients, carers, social care users (who may be older people or people with additional needs)
  - This will include whether the item is relevant and important for them currently in the context of being someone with a health condition, undertaking an informal carer role or using social care services.
- Compare modified items and questionnaire to the version 1 items and questionnaire.
- Test the interpretation of the order of the frequency response options as required for valuation.

**In advance of the Interview**

- Please see the intended item meaning for each item in the document
- Looking at the EQ-HWB intended meaning of items document will allow for a better understanding of how we hope responders will interpret the items. When a participant then talks about what they think about a draft item, it will be apparent to you during the session whether the item has been understood as intended.
- Prepare some useful general probes, for example:
  - *Can we just think about whether this is relevant for you?*
- It can be useful to remind participants the day before providing reminder details of how to attend (aligned to ethics)

**During the Interview**

- When first discussing the draft items, do not state the name of question, neither should you mention any of the examples. Instead, consider saying “*look at the question number 1*” (for example)
- Probe participants for examples, i.e., to provide context to their views, experiences, and opinions.
- It is important to elicit data for each component of an item (e.g. understanding and relevance).
- A distress protocol [not included here] will be initiated should participants become upset
- Ensure recording occurs in line with ethical approvals
- For online interviews remind participants that it is important to have their camera on throughout the interview.
- For in person interviews ensure appropriate interview space with due consideration to attendees needs, ability to hear clearly, privacy and comfort/welcome (e.g., refreshments)

It is assumed here that consent will be collected in advance in line with local ethics agreements.

The process for reimbursement is not included in this topic guide.

**Key:** Italics in blue denotes words for interviewer to use.

**Introductions and welcome**

- Introduce interviewer and their role
- Remind participants that the session is being recorded.
- Remind participants that if there is something that they do not want to discuss, that is ok. For example, you can tell them they can simply say they would prefer to pass on that question.

**Warm up and focusing the future discussion**

- To help frame the discussion to ‘health’ or ‘carer’ or ‘social care’ related aspects of quality of life ask the participant to start thinking about how the relevant aspect impacts on their life

*Before we get started on the questionnaire could you tell me a little bit about how [your role as a carer / your health] impacts on your life.*

*We have developed a questionnaire that is designed to measure the quality of life of adults with health conditions or people with additional needs, and for people who are caring for people with additional needs.*

*We would like to find out what people think about different ways that we can ask the questions.*

*There’s no right or wrong answer with anything I ask you today. We are really interested to hear your views and about your experiences.*

***(U=understanding, R=Relevance)**

| **Component to be tested** | **Wording and probes** | **What are we testing?** |
| --- | --- | --- |
| Interpretation of frequency response option order | *Please have a look at the words on the cards in front of you and place them in the order that you think they should come in.*   - If the interview is conducted online then the respondent will need a platform (such as wooclap) to enable them to sort the cards. - The cards will include all frequency options (none of the time, a little of the time, only occasionally, sometimes, often, most or all of the time).   *Thank you for doing that. Were there any cards that you thought were really similar or should really come in the same position?* | Response option order |
| Completion instructions | *Take a look at the questionnaire, specifically at the instructions on the top of page number 1, which start with the words “xxx”. Could you explain, in their own words, what you feel the instructions are asking you to do.*   - If issues with the recall period of 7 days are raised, encourage participant to provide examples (e.g., provide context as to whether the time period is considered suitable).   . | U |
| DIFFICULTY QUESTIONS FOR MODIFIED VERSION | | |
| Question 1 (doing day-to-day activities? (*e.g. working, shopping, housework*)) | *Now look at question 1. What does this question mean to you?*   - Encourage participants to answer in a way that can provide evidence to support understanding the item concept. i.e., encourage examples (e.g., context). - Probe if there are additional words that could be included in the example. i.e., encourage examples (e.g., context). - Probe if any of the words and examples are unclear and/or redundant. i.e., encourage examples (e.g., context). - Additional probes that could be considered if participants were not able to fully describe what the item meant:   - *‘What would an example of ‘moderate difficulty* doing day-to-day activities*’ be like? Could you give an example?’* | U |
|  | *We’d like to know whether this question would be relevant to ask when thinking about how your health/caring role impacts on your life. When you look at this question, what sort of things are you thinking about, with respect to your own health or caring role?* | R |
| Question 2 (getting around inside or outside? (*using e.g. a walking stick or wheelchair if you normally use them*)) | *Now look at question 2. What does this question mean to you?*  *[Note: do not read the question to them or highlight that the stem should be used]*   - Encourage participants to answer in a way that can provide evidence to support understanding the item concept. i.e., encourage examples (e.g., context). - Probe if there are additional words that could be included in the example. i.e., encourage examples (e.g., context). - Probe if any of the words and examples are unclear and/or redundant. i.e., encourage examples (e.g., context). - Additional probes that could be considered if participants were not able to fully describe what the item meant:   *‘What would an example of ‘a lot of difficulty* getting around inside or outside*’ be like? Could you give an example?’* | U |
|  | *We’d like to know whether this question would be relevant to ask when thinking about how your health/caring role impacts on your life. When you look at this question, what sort of things are you thinking about, with respect to your own health or caring role?* | R |
| VERSION 1 & COMPARISONS - DIFFICULTY QUESTIONS | | |
| Question 1 (How much difficulty did you have getting around inside and outside? *(using e.g. a walking stick or wheelchair if you normally use them)* | *Can you have a look at this slightly different way of asking the question – from your point of view does it seem to be asking anything different when its asked like that?*   - *Probe about the ‘and’ versus ‘or’* | U&R |
| Question 2 (How much difficulty did you have doing day-to-day activities? *(e.g. working, shopping, housework)* | *Can you have a look at this slightly different way of asking the other question – from your point of view does it seem to be asking anything different when its asked like that?*   - *Probe about using the stem versus not using the stem* | U&R |
| Differences between versions | *Order change*  *You’ll see in the first questionnaire the question about mobility came second, but it came first in the second version – do you think that makes any difference to how you would feel about filling it in?*  *Response option change*  *You’ll see in the first questionnaire the response answers use the response ‘some difficulty’ rather than ‘moderate difficulty’. Do you think these are the same or different?*  *Which of these versions do you like best?*   - *Probe as to why* | U&R |

| FREQUENCY QUESTION FOR MODIFIED VERSION | | |
| --- | --- | --- |
| QUESTION 3 (feel exhausted) | *Now look at question 3. What does this question mean to you?*   - Encourage participants to answer in a way that can provide evidence to support understanding the item concept. i.e., encourage examples (e.g., context). - Probe if there are additional words that could be included in the example. i.e., encourage examples (e.g., context). - Probe if any of the words and examples are unclear and/or redundant. i.e., encourage examples (e.g., context). - Additional probes that could be considered if participants were not able to fully describe what the item meant:   *‘What would ‘feeling exhausted most or all of the time’ be like? Could you give an example?’* | U |
|  | *We’d like to know whether this question would be relevant to ask when thinking about how your health/caring role impacts on your life. When you look at this question, what sort of things are you thinking about, with respect to your own [health/caring role]?* | R |
| QUESTION 4 (feel lonely) | *Now look at question 4. What does this question mean to you?*   - Encourage participants to answer in a way that can provide evidence to support understanding the item concept. i.e., encourage examples (e.g., context). - Probe if there are additional words that could be included in the example. i.e., encourage examples (e.g., context). - Probe if any of the words and examples are unclear and/or redundant. i.e., encourage examples (e.g., context). - Additional probes that could be considered if participants were not able to fully describe what the item meant:   *‘What would it be like to ‘feel lonely’ a little bit of the time? Could you give an example?’* | U |
|  | *We’d like to know whether this question would be relevant to ask when thinking about how your health/caring role impacts on your life. When you look at this question, what sort of things are you thinking about, with respect to your own [health/caring role]?* | R |
| QUESTION 5 (difficulty concentrating or thinking clearly?) | *Now look at question 5. What does this question mean to you?*   - Encourage participants to answer in a way that can provide evidence to support understanding the item concept. i.e., encourage examples (e.g., context). - Probe if there are additional words that could be included in the example. i.e., encourage examples (e.g., context). - Probe if any of the words and examples are unclear and/or redundant. i.e., encourage examples (e.g., context). - Additional probes that could be considered if participants were not able to fully describe what the item meant:   *‘What would it be like to ‘have difficulty concentrating or thinking clearly a little bit of the time? Could you give an example?’* | U |
|  | *We’d like to know whether this question would be relevant to ask when thinking about how your health/caring role impacts on your life. When you look at this question, what sort of things are you thinking about, with respect to your own [health/caring role]?* | R |
| QUESTION 6 (feel anxious) | *Now look at question 6. What does this question mean to you?*   - Encourage participants to answer in a way that can provide evidence to support understanding the item concept. i.e., encourage examples (e.g., context). - Probe if there are additional words that could be included in the example. i.e., encourage examples (e.g., context). - Probe if any of the words and examples are unclear and/or redundant. i.e., encourage examples (e.g., context). - Additional probes that could be considered if participants were not able to fully describe what the item meant:   *‘What would it be like to ‘feel anxious’ sometimes? Could you give an example?’* | U |
|  | *We’d like to know whether this question would be relevant to ask when thinking about how your health/caring role impacts on your life. When you look at this question, what sort of things are you thinking about, with respect to your own [health/caring role]?* | R |
| QUESTION 7 (feel sad or depressed) | *Now look at question 7. What does this question mean to you?*   - Encourage participants to answer in a way that can provide evidence to support understanding the item concept. i.e., encourage examples (e.g., context). - Probe if there are additional words that could be included in the example. i.e., encourage examples (e.g., context). - Probe if any of the words and examples are unclear and/or redundant. i.e., encourage examples (e.g., context). - Additional probes that could be considered if participants were not able to fully describe what the item meant:   *‘What would it be like to ‘feel sad or depressed’ often? Could you give an example?’* | U |
|  | *We’d like to know whether this question would be relevant to ask when thinking about how your health/caring role impacts on your life. When you look at this question, what sort of things are you thinking about, with respect to your own [health/caring role]?* | R |
| QUESTION 8  feel you lacked control over your day-to-day life? *(e.g. you had no choice over what you did or how things were done for you*) | *Now look at question 8. What does this question mean to you?*   - Encourage participants to answer in a way that can provide evidence to support understanding the item concept. i.e., encourage examples (e.g., context). - Probe if there are additional words that could be included in the example. i.e., encourage examples (e.g., context). - Probe if any of the words and examples are unclear and/or redundant. i.e., encourage examples (e.g., context). - Additional probes that could be considered if participants were not able to fully describe what the item meant:   *‘What would it be like to ‘feel you lacked control over your day to day life’ sometimes? Could you give an example?’* | U |
|  | *We’d like to know whether this question would be relevant to ask when thinking about how your health/caring role impacts on your life. When you look at this question, what sort of things are you thinking about, with respect to your own [health/caring role]?* | R |

| FREQUENCY QUESTION ON VERSION 1 and COMPARISON | | |
| --- | --- | --- |
| QUESTION X (Concentrating thinking clearly) | *Now look at question X. What does this question mean to you?*   - Encourage participants to answer in a way that can provide evidence to support understanding the item concept. i.e., encourage examples (e.g., context).   *‘Does ‘having difficulty’ seem like a similar or different question to ‘having trouble’?*  *‘Which version of the question do you prefer?’*  *‘Can you tell me why you prefer that one’?* | U |
| QUESTION X  (Control) (feel you had no control over your day-to-day life? *(e.g. had no choice to do things or have things done for you as you liked and when you wanted))* | *Now look at question X. What does this question mean to you?*   - Encourage participants to answer in a way that can provide evidence to support understanding the item concept. i.e., encourage examples (e.g., context).   *Probes*  *‘Does the way the explanation is given in this question seem similar or different to the previously worded question.’*  *Which do you find easiest to understand?*  *‘Does asking about feeling you have ‘no control’ differ to ‘feeling you had a lack of control’?*  *Which do you find easiest to understand?* | U |
| Differences between versions | *Response option change*  *You’ll see in the first questionnaire the response answers use the response ‘a little of the time’ rather than ‘only occasionally’. Do you think these are the same or different?*  *Which of these versions do you like best?*  *Probe as to why* | U |

| PAIN QUESTION FOR MODIFIED VERSION | | |  |
| --- | --- | --- | --- |
| QUESTION 9 Pain: MODIFIED  Please choose **one** answer to describe how much physical pain you had in the **last 7 days**. Did you have:  No/Mild/Moderate/Severe/Very severe pain | *Now look at question 9. What does this question mean to you?*   - Encourage participants to answer in a way that can provide evidence to support understanding the item concept. i.e., encourage examples (e.g., context). - Probe if there are additional words that could be included in the example. i.e., encourage examples (e.g., context). - Probe if any of the words and examples are unclear and/or redundant. i.e., encourage examples (e.g., context). - Additional probes that could be considered if participants were not able to fully describe what the item meant:   *‘What would an example of ‘moderate pain’ be like? Could you give an example?’* | U | |
|  | *We’d like to know whether this question would be relevant to ask when thinking about how your health/caring role impacts on your life. When you look at this question, what sort of things are you thinking about, with respect to your own [health/caring role]?* | R | |

| COMPREHENSIVENESS OF MODIFIED VERSION | | |
| --- | --- | --- |
| Wrap up (considering the whole EQ-HWB-S questionnaire) | *Having looked at all the questions, when you think about the impact of [health/your caring role] on your life is there anything missing? Are there any other questions that you think we should we have included that are not currently there?*   - Establish whether any new suggestions relates to any specific health condition or need - Encourage participant(s) to give examples (i.e., context). | Comprehensiveness |
|  | *This is designed to be a short questionnaire - is there anything in there that you don't think is important or where you think we have questions that are a bit repetitive?* | Redundancy / Lack of relevance |
|  | *Finally, do you have any other comments or suggestions to feedback on about the questionnaire or any of the questions we’ve looked at today?* | Comprehensiveness |

**Closing of the interview**

Explain that this is the end of the discussion.

Thank the participants for their time – provide participants with an opportunity for a debrief discussion on how answering these questions has made them feel.

Explain when participants can expect to receive their reimbursement.

## Supplement 3: Coding framework

| **Understanding or interpretation correct including value of examples** | Respondent understands and interprets the item correctly |
| --- | --- |
| U1Wrong or ambiguous interpretation | Respondent interprets the item differently than what was intended by the developers |
| U2 Difficulty interpreting/ odd wording | Respondent comments that they are unfamiliar with terms/phrases or struggles with a complicated structure. Respondent finds terms/phrases unusual or odd. |
| U3 Narrow interpretation | Respondent focuses on one aspect of the construct or are unsure about the focus of the item |
| U4 Word means a different thing in translation* | Word used in translation does not match the intended meaning |
| U5 Different answers for different aspects of items | Respondent has difficulty with double-barrelled items |
| U6 Issues with examples | Respondent has difficulty with examples used and interpret the item incorrectly as a result |
| Unclear | Unclear if respondent understands |
| **Relevance of item** |  |
| R1 Item not relevant | Respondent reports that a question is irrelevant/ inappropriate and should not be asked. This should be contextualised appropriately i.e. if a mixed population is used it should be clear which group thinks this e.g. carers |
| R2 Respondent says item not relevant because they misunderstood item | Respondent reports the item is not relevant, but they misunderstood the question therefore relevance is not clear. |
| Unclear |  |

*Note that concerns over translation are not relevant in the UK context, but the same core coding framework was applied to all related international studies.

## Supplement 4: Reflective considerations

The interviewers were conducted and coded by two young female researchers (VG and AM) who have not had any prior involvement with the development of the EQ-HWB. It is possible that participants of a different gender may have felt less comfortable disclosing information relating to their health and wellbeing. However, the style of interview relying mostly upon verbal probing and interpretation of items is less personal than a predominantly ‘think-aloud’ approach.

The other members of the research team (CM, TP, and JC) were involved in the EQ-HWB development and may therefore have a vested interest in demonstrating that the instrument is performing well. However, preferences between original versions and potential modifications, which form a substantial component of the research, are neutral in this respect.

The interviews were conducted by a medical doctor and health economist (AM). This dual background supported engagement with participants on both health and wellbeing concepts, while also introducing the possibility of interpreting responses through professional and technical lenses. All interviewees were older adults with medical conditions, which facilitated rapport but also introduced differences in lived experience. Within the research team, the interviewer was the least experienced member but had autonomy to adapt the interview process and analysis. This reduced the influence of senior colleagues’ perspectives or biases and helped ensure that participants’ voices remained central.

## Supplement 5: Tables

## Table S1: Description of Participants (n=26)

|  | **N** | **%** |
| --- | --- | --- |
| **Female** | 13 | 50 |
| Age: Mean (SD), range | 53 (SD 1.36), 24-68 years | |
| **Ethnicity**: White | 23 | 88 |
| **Education**: Bachelors degree or above | 17 | 65 |
| **Employment**: In employment/self-employed | 13 | 50 |
| **Long term condition** (*Do you have any long-standing physical or mental impairment, illness or disability which has been diagnosed by a doctor? By 'long-standing' we mean anything that has troubled you over a period of at least 12 months or that is likely to trouble you over a period of at least 12 months)* | 26 | 100 |
| **Informal carer** (“*Is there anyone (family or friend), either living with you or not living with you, who is sick, disabled or elderly whom you look after or give special help? Please exclude paid caring work.*”) | 6 | 23 |
| **Social care users** (*Have you used social care services or support for yourself over the last 6 months (e.g. home care, home help, Personal Assistant, day-centre, residential care or nursing care home)?*”. | 2 | 8 |
| **Health conditions (patients may report more than 1)** |  |  |
| Asthma | 5 | 19 |
| Arthritis | 10 | 38 |
| Cancer or malignancy | 1 | 4 |
| Coronary heart disease | 1 | 4 |
| Congestive heart failure | 2 | 8 |
| Diabetes | 3 | 12 |
| Epilepsy | 1 | 4 |
| Heart attack or myocardial infarction | 1 | 4 |
| High blood pressure/hypertension | 4 | 15 |
| Multiple Sclerosis | 1 | 2 |
| Other long standing or chronic condition | 12 | 46 |
| None of these | 3* | 12 |
|  |  |  |
| **Duration of the longest condition reported (mean)** | 29.26 years |  |
| **Carers hours per week spent caring** |  |  |
| 1-19 | 3 | 12 |
| 20-49 | 1 | 4 |
| 50+ | 1 | 4 |
| Missing hours | 1 | 4 |
|  |  |  |
| **Health overall** |  |  |
| Very good | 1 | 4 |
| Good | 8 | 31 |
| Fair | 12 | 46 |
| Bad | 4 | 15 |
| Very bad | 1 | 4 |
|  |  |  |
| Life satisfaction (0-10), Mean (SD) | 6.67 (SD 1.78) | |

*Note that all participants reported having “long-standing physical or mental impairment, illness or disability which has been diagnosed by a doctor?”. It is therefore assumed that these participants have a disability or impairment they do not consider to be a health condition.*

## Table S2 Quotes for item understanding in EQ-HWB-9

(see Supplement 3 above for an explanation of the codes applied)

| **Item** | **Demonstrating understanding aligned to intended meaning** | **Demonstrating concern with understanding** |
| --- | --- | --- |
| Activities | - *“That's just about everything that you do in a normal day. Particularly as I don't work, it's all to do with either doing surveys, housework, shopping, which I do once a week, a bit of cleaning.” (AM01 M60+)* - *“so how much my health has affected me when trying to do day-to-day activities. Just normal stuff. ... So, I just think like school run, getting kids ready for school, cooking, food shopping, cleaning, driving” (V06 F1839)* | 100% understand as expected |
| Getting around | - *“So I assume that that means your kind of mobility in your area. So the examples you give using a walking stick or wheelchair if you normally use them. And yes, I would say that that's kind of going from room to room in my house. I have stairs… that it's about how I would get around my normal kind of location where I live and whether… measuring that against the scale.” (AM02 M4059)* - *"So how has my mobility been both at home and when I go out? " (VG07 F60+_C)* | - U3 (Extreme mobility problems considered only): *“I imagine that that's the kind of situation where you might require a walking stick […] so obviously this is I guess a slightly more extreme version of the previous question because it's seeing if your mobility is restricted in any major way.* (AM06 M4059) - U6: (Issues arose because of the examples) *"If I had to be with a wheelchair or with some sort of walking aid, that would be in that case. So, that's what I see there when I see that that type of thing... is the times that I've been in a wheelchair is when I've been hospitalized"* (AM04 M4059_C) - U6: (Issues arose because of the examples) *“No difficulty. I don't use a walking stick. I'm not in a wheelchair. So for me it's not particularly relevant but I understand in that case I'd say no difficulty.”* (AM08 M60+) - U6: (Issues arose because of the examples) *"So I don't use a walking stick or a wheelchair. But if somebody did, then that is a relevant question for somebody who has that restriction in mobility."* (VG08 M60+) - U6: (Issues arose because of the examples) *"It's asking if I can walk without a stick or wheelchair"* (VG11 M60+) - U6: (Issues arose because of the examples) *"That's basically asking do I need to use anything, isn't it? So you know a walking stick which I do use from time to time."* (VG12 M60+) |
| Exhausted | - *“So, to me, exhaustion can manifest itself in lots of different ways. It could just simply be the lack of motivation to do something to just being utterly depleted of energy. So, there's a real scale really on exhaustion. ...I think it's quite a cliche when someone says, I'm totally exhausted." Because to me exhaustion, is being completely depleted of energy. So just no motivation to do anything. Just tired, lethargic, just needing to sleep.” (AM05 F4059_C)* - *"exhausted feels more extreme than tired" […] "most people have felt tired at some point each day. But exhausted is you just feel completely drained and like everything is hard to do" (VG02 F1839)* | 100% understand as expected |
| Lonely | - *'"I think loneliness is not just about physical solitude. It can also be just feeling isolated from people around you. you can be lonely and yet you can be in a group of people. So, it's about being disconnected possibly" (A05 F4059)* - *"I interpret that as wanting to be around other people but not being able to see people when I want to. So being alone and being lonely are not the same thing. One can be alone and not lonely, but one can be around people but still be lonely if you're around the wrong people." (V03 M1839)* | 100% understand as expected |
| Difficulty concentrating / thinking clearly | - *“I would say that's feeling foggy or having to read things several times for it to make sense and maybe it still doesn't make any sense or easily distracted that you reading something and then something else. And so you can't fully read, a sentence or a paragraph a page of something without being distracted” (AM08 M60+)* - *"Like brain fog which I think difficulty concentrating or thinking clearly. I have memory lapse. when you're so overwhelmed with medical stuff, it's very hard to focus on anything else. So it's hard to think clearly outside of that" (V06 F1839)* | 100% understand as expected |
| Anxious | - *“Do you feel uptight.. worried? Do you have panic attacks? Do you have fear? I think the biggest thing about anxiety is fear. for me it is I would say” (AM10 F60+)* - *"Worrying about things particularly worrying about things more than they deserve. Letting the worry prey on your mind and so that it affects your quality of life." (VG10 F60+)* | 100% understand as expected |
| Sad/depressed | - *“So is that feeling that kind of.. linked to being melancholy… to having a kind of negative view on yourself or on the world. And it has sad as the kind of antonym for happy and so it's the inability to find joy in every day.” (AM02 M4059)* - *"I believe being sad is just you have feeling emotionally down. Whereas depression is literally mental health where you can't control it. When someone is depressed because I've seen people go through it. They can't seem to break out of it. They stay in bed all day. They don't want to talk to anybody. They don't want to do anything. And it's not because they're lazy or because they're being a Debbie Downer. They physically cannot move. It's like there's a weight on them. " (VG05 F1839)* | 100% understand as expected |
| Lacked control | - *"that could be that someone was making decisions on your behalf or someone had intervened in the decisions that you'd already made. And the lack of control could be, something simple like changing your routine or giving you a food that maybe you don't like. Or it could just be something a lot more serious." (AM05 F4059)* - *“I used to be quite independent when I was younger, but now nowadays I'm pretty reliant on people around me because I probably couldn't handle life by myself. I probably need some level of help whether it's just taking care of my daily chores or that sort of thing" (VG04 M1839_C)* | - U2 (has difficulty with interpretation): *"there are some circumstances where yeah I don't really have a choice but to do something […] as in when I say that I mean objectively you don't have a choice but you have to do something but there are times when you do actually have a choice but you might not realize it. […] Okay so let's say for example okay I work remotely two days a week but for whatever reason if I need sometimes my work might say okay I have to be in office on this day because there is something happening [...] In such a situation it's objectively I'm being told do this right I'm basically given an order and so I sort of don't have control over my life but then again you could argue I could still say no and still stay at home and maybe face the consequences" "But you sort of feel like you're being pressured to do one option. […] But then at the same time there may be just certain other less concrete cases where maybe just due to the state that someone's in they might not notice all the possibilities and they might feel like they have to do a certain thing a certain way because it just didn't cross their mind that there are other ways to go about it." […] "if there is some situation where you feel like this is only choice but you haven't realized it if you haven't realized it then you don't actually know […] So this question makes me wonder which of these sort of two possibilities it's aiming at and I'm not actually sure."* (VG03 M1839) |
| Pain | - *For me, mild is niggles. you get out of bed and you get a bit of pain with your knees perhaps as you get up or your back whatever, but it doesn't stop you doing whatever you're doing. And if you choose to take, you wouldn't even choose to take paracetamol or something like that to get rid of it because it's negligible. A severe pain it stops you doing what you want to do. You're in too much pain to actually move around or do things” (AM08 M60+)* - *"So how much pain in your body, like actual pain, like something you'd need to take painkillers for" (VG06 F1839)* | - U1: *"This one I think is a bit ambiguous. […] I look at that and I don't know if we're sort of trying to estimate like what proportion of the time have I been in pain or how severe has the pain been? Er because they're using words like no, mild, moderate, severe, very severe. But then like what if I have mild pain but it's chronic and it never goes versus I fell and I broke my ankle, so I had very severe pain but only once, right?"* (VG02 F1839) - U1: *“"**they talk about mild, moderate, very severe but there are two factors here. One the intensity and two is the frequency and it doesn't distinguish between them. So, if I have a really bad headache for one day, if I have a really bad headache for one day is that severe? I don't know. Or is that mild because it's only one day or as I mentioned, I just went to the gym, so my whole body feels sore for three days. Is that moderate because that's almost half the time even though the soreness is not really affecting my day-to-day life."* (VG03 M1839) - U1: *"It's not like being in severe pain,* *which for me means it's there a lot of the time. Moderate pain is for me sort of transient"* (VG13 F60+) - U1: *“**Moderate physical pain is to me it's a frequent pain that I've become used to. It's like I could have a bout of this or sort of continuous to someone else it would be terrible but because it's been going on for so long.”* (VG14 M60+) |

*Each participant is represented by a participant ID based on Interviewer initials (VG/AM), interview number, sex (F/M), age group (1839, 4059, 60+), and whether they are also a carer (C) e.g. VG04 M1839_C*

*Where multiple quotes demonstrate understanding as aligned to the intended meaning only two are shown; for concerns with understanding all cases are shown.*

## Table S3 Quotes for item relevance for EQ-HWB-9

| **Item** | **Relevant** | **Not relevant** | **Relevance Unclear** |
| --- | --- | --- | --- |
| Activities | - *“As I said that things like just getting ready takes longer because I get in the shower, but I'm holding on to the wall […] it's definitely affected day-to-day activities […] if I cleaned and then I felt absolutely exhausted. It sort of put me out for the next day things like that. Driving... My driving's affected because my foot feet are numb.” (AM11 F60+)* - *“Yes. Well because of the discomfort I have of it, usually the fatigue and the aching, it makes I can work up the energy. It can even make the activity quite difficult to do because I'm sore or whatever." (VG04 M1839_C)* | None judged not relevant | *Interviewer: Do you feel as though your health condition impedes any of those things? Participant: “No, it doesn't.” (AM08 M60+)* |
| Get around | - *" I use crutches, so I do have difficulties. and some days I have more difficulties than others. So, I would give an average because it's asking for the seven days. I would give an average of where I was on that scale across the seven days.” (AM03 F4059)* - *“That's just everyday living. That's just what I do. Yeah. Getting around inside, I don't use a stick. I use walls, furniture, anything I can grab my hand on. Outside, I can go a very short distance, about four or five steps without anything, but it's a bit dangerous. I use an elbow crutch all the time. So yeah, that is very relevant. " (VG14 M60+)* | - *R1 “I'm thinking about my mom … and the limited sort of mobility that mom has now when she has gone out she's sort of unsteady on her feet. So she doesn't use a walking aid but still has that difficulty getting around. [not relevant to the participant]” (AM05 F4059_C)* - *R1 “in terms of myself, I don't have any of those particular problems [extreme mobility issues]. So, I'm not sure I could relate to that.” (AM06 M4059)*   *AR1 “So I have no physical difficulty physically moving and walking…” (VG03 M1839)*   - *R2 “that would be severe asthma attack with hospitalization. If I had to be with a wheelchair or with some sort of walking aid, that would be in that case. So, that's what I see there when I see that that type of thing... is the times that I've been in a wheelchair is when I've been hospitalized” (AM04 M4059)* - *R2 "Personally speaking, I don't have such a problem with mobility as walking with a stick or a wheelchair.” (VG08 M60+)* - *R2 "I don't need to be asked that question." "I don't need any of those things. Thankfully. Yeah, I can get about.” (VG11 M60+)* - *R2 “"Again I think it's a relevant question. Thinking of my personal situation when I'm out sometimes I do need to use a walking stick. I don't use a wheelchair. I don't need that. Sometimes when I'm walking around the house, if I suddenly get hit with pain, then I might need to lean on furniture just to maintain my balance." (VG12 M60+)* - *R2: “no difficulty.* *I don't use a walking stick. I'm not in a wheelchair. so for me it's not particularly relevant. but I understand in that case I'd say no difficulty.” (AM08 M60+)* | *“100% yes. Yeah, absolutely...in my house, I have stairs that it's about how I would get around my normal kind of location where I live and whether measuring that against the scale” (AM02 M4059) [General unclear if specific to their own health]*  *"but the second one a little bit less cuz erm I mean if you count let's say if you count distractions right in inside the house and it also sort of applies to the second question but I know that's not what the second question implies. Cuz there are distractions where I go and I'm not really getting from point A to B. I'm rather going from A to point A point A, to A point B, then B." (VG01 M1839)* |
| Exhausted | - *In terms of me, I know that my mom feels exhausted as part of her condition, but then I do as well because sort of I'm juggling a job, a family, and I also live six miles away from my mom. So, it's the constant back and forth and something as simple as my mom just needs a pint of milk from the shop for example yesterday and me having to travel effectively 12 miles to drop off that shopping. So exhaustion can be not only from the patient but also from the carer I would say” (AM05 F4059_C)* - *"I have a young daughter, one and a half year old daughter. I'm not really safe to be alone with her erm because the fatigue can hit quite quickly erm so I can be alone with her in a room …I have to be really careful in pacing my energy each day" (VG02 F1839)* | - *R1 "Not really. No, cuz I don't get to that touchwood. I don't get to that stage yet." (VG11 M60+)* | *“… but I would get tired if I'm exercising. or I guess if I don't have a good night's sleep, you would feel tired. there are occasions I don't sleep well and therefore you get up in the morning just feeling tired. you don't particularly want to do an awful lot, but you may have to sort of rest up a bit and may even, if you haven't slept well and you're able to is have an afternoon siesta […] I don't get that and therefore I would get exhausted if I go on a fast…. I wouldn't get exhausted. I don't tend to get exhausted” (AM08 M60+)* |
| Lonely | - *“you know how I explained the three different types of loneliness there. Sort of a social loneliness or an emotional loneliness or a physical loneliness as well….It's for me I would say an emotional loneliness. I mean maybe the word for that is isolation. I don't know. feel isolated. yeah,… (AM03 F4059)* - *“Yeah, quite relevant a lot of it...lonely yep…You can't join in and because I don't get out so much because that in itself's a bit of an issue. I do spend a lot of time on my own. Yeah, I'm feeling lonely because pretty much am." (VG14 M60+)* | - *R1 “It's not something I suffer from personally, particularly in the connected world we live in now. I can always find somebody to connect with if I need to. But I've never been one to feel I need anything more than my own company” (AM01 M60+)* - *R1 “I don't know if it's particularly relevant to my own. I have a family so I'm very very infrequently actually lonely.” AM06 M4059)* - *R 1 “I never do feel lonely so it's hard to say…but if I wanted social contact my health conditions don't prevent it at all.” (AM09 M60+)* - *R1 “I've got plenty of family and friends around me. So, … I don't feel lonely. Sometimes I quite enjoy a bit of alone time.” (AM12 F60+)* - *R1 "Feel lonely, oh I never feel lonely." (VG07 F60+_C)* - *R1 "For me, no. Firstly, I'm an introvert, so I rather like being on my own. And secondly, I wouldn't say I was not quite a social influencer, but I'm a popular science writer on [website]. I have more friends than I can keep up with." (VG10 F60+)* | *“for me I do not have regular interaction during a working day with people. So you start at 8 or 9:00 and you'll go through 4 or 5:00 and you'll see no one. So yes, but it's not my health condition as such cuz my health condition is control. But it is a mental health condition. I guess I got to think of it in those terms. (AM08 M60+)*  *“But bear in mind that I'm generally okay in my own company most of the time. So, I don't often feel lonely." (VG03 M1839)*  *“I say it's relevant. I say of everything listed, it's probably the one that affects me least. But that's only because of the internet. if we were 50 years in the past, then no, I'd be very lonely. …I have friends I'm able to talk to very frequently." (VG04 M1839)* |
| Difficulty concentrating / thinking clearly | - *“for me, I can get stuck in a loop sometimes of thinking the same thing over and over again sometimes or just getting started on activities when I can't concentrate. Definitely this applies to that unfortunately.” (AM07 F4059)* - *"Yes, I think it possibly can do because I sometimes have to, or I don’t have to, but I sometimes focus on the pain and discomfort that I'm in. So whatever I might be doing at the time, I may not be concentrating fully on that. I may not be thinking clearly, might as a result make wrong decisions about things. I don't know. So again, yes, I would think that that would be something that would be relevant." (VG12 M60+)* | - *R1 “No, not for me. I don't have… I guess question five leads into question three - when you feel exhausted you don't think clearly, you have a foggy mind if I haven't slept some nights then things take longer to think about when you do it but overall it's not an issue for me. I also have dyslexia so reading things tends to take longer for me. But I wouldn't say it affects my life personally” (AM08 M60+)* - *R1: “No, I think I manage my health condition really well. So, I don't have any problems concentrating.” (AM12 F60+)* - *R1: "I'm very lucky I didn't get brain fog, but a lot of people do with long covid or just with age that people just kind of go off the point. For me, it's just because I get tired" (VG10 F60+)* |  |
| Anxious | - *“I'm always aware of my heart condition. and if I get pains or anything, I sort of double check that it's not my heart, which hasn't been, and it's a muscular thing. So yeah, I'm very aware of occasions of anxiety to do with mostly my heart..” ((AM08 M60+)* - *"And I suppose for me the two sources of anxiety either things like that or over my health really. For example, this latest diagnosis of hyperparathyroidism, if it doesn't get sorted out, could have quite serious effects on my heart and my bones. So, I will start thinking about that and worrying about that" (VG11 F60+)* | - *R1 “I mean I don't feel anxiety through my health conditions […] it's more …I mean I take in news media 24/7. (AM09 M60+)* - *R1 "I have anxiety about my landlord, but that's not an inappropriate anxiety, let's say." "No, not really. Me? No, because generally speaking, when I'm not having to deal with something like that, I'm not an anxious person. " (VG10 F60+)* | *“I think health conditions do make you feel anxious and edgy. So, it's got relevance.” (AM03 M1839)* [note the comment here is only around general relevance rather than the participant talking about their own experience] |
| Sad/depressed | - *“And that's definitely relevant because, I think chronic pain is depressing. any chronic condition is very draining, wearing, and can leave you feeling sad. “(AM03 F4059)* - *"I do have a bit of a history of depression …For me, I think it is still relevant. it is still something that's there. It's almost like a bit of a backdrop. " (VG13 F60+)* | - *R1 “no, the only way I can answer that question is no. I'm being pretty upbeat. we haven't long returned from holiday. We've been on a three-week cruise and life's pretty good and…” (AM12 F60+)* - *R1 " it's not relevant to me, but I can see lots of reason why it could be relevant to people who are dealing with social care or sickness" (VG09 F4059_C)* | *"Depression, no. Sad, yes, because lot of people that I have died. Or a lot of people that I love are in difficult or abusive situations so I’m sad about that. I'm Sad about things like war and the earthquake in Myanmar. I've got family out there. " (VG10 F60+)* |
| Lacked control | - *“I think sometimes I feel I lack control because I've not been terribly good at overcoming my anxiety or my worry. and because I can't seem to overcome the situation, I feel that I don't have much control in my life” (AM10 F60+)* - *"Yeah, pretty much. Most of all the time. I used to be quite independent when I was younger, but now nowadays I'm pretty reliant on people around me because I probably couldn't handle life by myself. I probably need some level of help whether it's just taking care of my daily chores or that sort of thing" (VG04 M1839)* | - *R1 “Personally, no” (AM01 M60+)* - *R1 “I don't think it does. This doesn't feel like it applies to me.” (AM06 F4059)* - *R1 “So, I feel as I've got total control over my life because I don't feel that I'm restricted by times as such.” (AM09 M60+)* - *R1 “And therefore, that may be a more pertinent question for people who are physically being cared for rather than somebody who was like myself or somebody who was in a wheelchair or walking with a walking stick who was more in control of their own day." (VG08 M60+) [not relevant for them as not disabled or being cared for]* - *R1 “in general, no, I live alone and make my own decisions except about the fact that I can't even knock a hole in the wall to put up a picture or put up shelves cuz I don't own the house, that's extremely frustrating. But other than that, no." (VG10 F60+)* - *R1 "No, I don't really feel that, but I mean, it's just a different life. I've got control over a different life to an ordinary life if you're with me. (VG14 M60+)* | *“…If someone else is erm making food, then I mean I don't know. You still have control though because you're still consenting to it. I don't know. I find this a tricky one to parse…” (VG02 F1839)*  *“there are some circumstances where yeah I don't really have a choice but to do something …But I don't know if that really counts as feeling not in control that that could be one thing where I feel like I lack control over my life cuz in a way I still do have a choice of whether to go in whether to actually do what I'm told or do what I want.” (VG03 M1839)* |
| Pain | - *“I'd probably be thinking about the neuropathy that I have in my feet and legs. and sort of the current issue that I'm suffering with frozen shoulder.” (AM06 M4059)* - *"I've had hip pain, in the past week once again it felt like I'd fractured my hip at one point. That was painful." (VG05 F1839)* | - *R1 "if we could swap the word pain for… it's really hard to describe… for discomfort. So, when you can't breathe, it's very difficult thing to describe. It's not what we describe as acute pain. it's discomfort, but every second is a struggle" (AM04 M4059)* - *R1 "Physical pain erm no …no not really I think mine hasn't given me any physical pain at all " (VG01 M1839)* - *R1 "In my case it was not relevant because it was more exhaustion and I had treatment but I have no physical pain" (VG09 F4059_C)* | *None judged to be unclear; one missing* |

*Each participant is represented by a participant ID based on Interviewer initials (VG/AM), interview number, sex (F/M), age group (1839, 4059, 60+), and whether they are also a carer (C) e.g. VG04 M1839_C*

*Where multiple quotes demonstrate relevance only two are shown; for concerns with relevance all cases are shown.*

## Table S4 Quotes for comprehensiveness (missing items) of EQ-HWB-9

| **Nothing missing** | **Missing items** |
| --- | --- |
| - *No, I thought it was very very thorough because it covers the emotional and the physical I think pretty adequately. (AM01 M60+)* - *There was nothing that jumped out that I thought That would be something I'd be expecting to answer. And there's no kind of extra level of detail that I think is missing here. (AM02 M4059)* | - *I mean you're looking at physical health,…emotional health, and I'm wondering about sort of spiritual health. So any questions about how health conditions impact on mind, body and spirit? (AM03 F4059)* - *Possibly in the last seven days did you feel like you needed support from someone else? I think that should be included because quite often with a carer they are the only port of call and sometimes the carer needs support in be something simple like the carer isn't feeling very well themselves and isn't feeling up to the task of caring for someone else. So I think being mindful of the carer being someone with human fallibility really that they can become ill and they can need that support is important. (AM05 F4059_C)* - *“some sort of questions about are you able to get access to health care or advice” (AM06 M4059)* - *I think you should ask a context question like "Have you recently experienced an event you would consider traumatic?" Because sometimes when you're filling in these kind of questionnaires, they make you feel really bad and then you need to sort of remember, yeah, and this happened to me, so I might have a really good reason why I feel all these things. And also if somebody was reading it, it would help them join the dots of why your quality of life is as it is. And I think it would help them to treat you. I don't think you can answer all these kind of questions with no context. (AM07 F4059)* - *Maybe something like, is there anything you could do to overcome this or is there anyone that can help you with this? That could be a question because it's like asking, your activities and your pain. Who can you go to for support? Do you feel you can support.. your doctor or whoever can help you? (AM11 F60+)* - *I suppose broader questions around how the doctors manage it and appointments and things like that (AM12 F60+)* - *"It's like an unlimited I don't know. I find these boxed questionnaires like putting you into categories and not everyone fits into a box it's just not life to fit into boxes. Maybe there should be a part that just maybe asks you to how do you feel like a specific part? "And I don't know, I'm just put off because of my PIP experience. These sort of boxes and tick boxes, I just don't have good feelings towards them. I feel like that really doesn’t represent, those nine questions could never represent the difficulty in my life. And I feel you have to speak to someone like a tick box is never going to be adequate. (VG06 F1839)* |

*Where multiple quotes demonstrate comprehensiveness only two are shown; for concerns with comprehensiveness all cases are shown.*

## Table S5 Quotes for comprehensiveness (redundant) of EQ-HWB-9

| **Nothing redundant** | **Redundant / Overlap** |
| --- | --- |
| - *"I think it's straight forward, not repetitive and it's different" (V01 M1839)* - *“No, I thought they were all important.” (A10 F60over)* | - *Maybe anxious and sad and depressed could be merged together. I think how often have you felt anxious, sad or depressed could be triadic as opposed to just having number six as it by itself. [….] Yeah, because I think anxiety and depression although they are different they're just as different as sadness and depression. So I think you can put all of that in the same bracket rather than having six and seven separate. That's just my thought. It's not necessarily right, but I think that would just if you have been anxious, you have probably been sad or depressed. And likewise, if you've been sad, you've probably been anxious. (A05 F4059)* - *I think question two is basically repeating question one, day-to-day activities. that could be incorporated in there because if you can do your day-to-day activities, then you're asking basically the same question in number two, getting around. So if you say you can do your day-to-day activities and you say no difficulty then you're going to say no difficulty to question two. Or you need to reword question two, I guess, are you able to do day activities with aids? but I still think it's fundamentally the same question. you're the researcher, so you may be trying to get something different out of it, but I don't see what you're trying to get different out of that. you're really looking at someone's mobility and day-to-day mobility (A08 M60over)* |

## Table S6: Themes and quotes supporting preferences for modification of layout – use of stem for first two items vs repeated text

| **Preference** | **Theme** | ***Quote*** |
| --- | --- | --- |
| Prefer stem (modified) (n=11) | **Readability** Faster reading (n=4) | - *“because I think it just makes sense to have it like this [stem included only once] because we've got the colon here in the last seven days. So I don't think it's necessary to have it worded in that way in every single question to have in the last seven days. I don't think it's necessary top one. I think it's exactly the same, but it just makes it maybe more speedy to read through in the top version. (AM05 F4059_C)* - *“**I think that is easier, clearer to read really than reading it again. I personally prefer the first one. it's just less reading really. (AM11 F60+)* - *"I don't believe it changes the meaning and I think I prefer it the way I first saw it because it seems a waste of effort to have to read it again. It's very obvious that both are preceded by the seven days question or part of the question." (VG07 F60+_C)* - *"Well you’re repeating yourself here and at the top there. I mean that personally speaking, I know exactly what that means. In the last seven days, how much difficulty have you had with A B C? 1 2 3 4 5. I get that. ""It only needs to be there once I think" (VG08 M60+)* |
|  | **Readability** Cleaner / more appealing (n=7) | - *“Oh, first one is much, much cleaner. I probably would use the first one. I probably wouldn't use the second one at all. It's a lot of bold and a lot of erm words. So yeah first when it comes through just explains it very clean" (VG01 M1839)* - *"I think I prefer the one where the in the last seven days is in the top left corner. It just feels a bit repetitive and wordy if you repeat in the last seven days in every single question" "I think the second one with the repetition feels a bit cluttered" (VG03 M1839)* - *"The top one is cleaner. It's easier to read because obviously the second one has a lot of redundant information. Not redundant so much, but it's less clean. It uses more words to say the same thing." (VG04 M1839_C)* - *“But if it's a short survey, having it in the top makes sense. It just looks better" (VG05 F1839)* - *"The top one is better if because it's neater and easier on the eyes if you only have a few headings under it. (VG10 F60+)* - *"I don't think it changes the meaning, but the top one is clearer. If it's done in a block like that, then I would say that the top one is the better one to go with." (VG12 M60+)* - *"I think it's asking the same question, but I would prefer the first one [not repeating]" "**I just think maybe it might just be aesthetically, but for my brain that's just, that’s less overwhelming than the second one…I do prefer the first one. [not repeating]" (VG06 F1839)* |
| Prefer original – full sentence (n=13) | **Appropriate** Reminder / emphasis on instructions (n=9) | - *“you've included the last seven days part into the whole, which I guess is helpful in a sense because it stops you having to keep referring back up the chain to make sure you're doing what you're supposed to be doing in the survey. So, I prefer the second one because it's emphasizing it's the last seven days we're talking about, not just generally” (AM01 M60+)* - *but when you put it all in one chunk, you think more about the seven days, but in the top one, you might forget that it's within a week (AM07 F4059)* - *I think it's good to remind people in the sentence that they're talking about the last seven days. Because you may read the top part and then you may go down to question one and think, " yeah, and forget it's the last seven days." But that actually reminds you, when they're asking that particular question. (AM10 F60+)* - *“I think having it there makes it less likely that people are going to miss it. People do skim questionnaires” (VG02 F1839)* - *"it focuses you because you might look and then go straight to the questions. So it reminds you to think just in the last seven days rather than think in general. So if you're for you the last seven days important the second option [repeating] is better" (VG09 F4059_C)* - *"For me, when I'm doing questions on prolific, doesn't have to be about health, just general surveys. If they're quite long, I sometimes forget and have to go back to the top to check is this within seven days or is this how I'm feeling right now or is this over the month? So I think it's possibly helpful to just repeat it I think because it just focuses. I mean if it was a really long one it might get a bit tedious if you kept saying the last seven days but if it's just the couple of things two or three things I think it's probably helpful to have it in the actual question. ""and keeping you focused. Oh it's just in the last seven days. I think so." (VG13 F60+)* |
|  | **Readability** Easier to read (n=4) | - *"To be honest I think you’ve got to do that. Yeah. Particularly with me, you've got to be obvious with what you're asking. You can't expect me to work out the question. Be obvious with it. It doesn't annoy me that it's repeated." (VG14 M60+)* |
| No difference (n=1) |  | - *“No, I don't see any real difference there. To be honest, both work equally well for me.” (AM09 M60+)* |
| Unclear (n=1) |  |  |

## Table S7: Themes and quotes supporting preferences for modification of layout order of items: activities first vs mobility first

| **Preference** | **Theme** | ***Quotes*** |
| --- | --- | --- |
| Prefer activities first (n=11) | **Appropriate** Easier item to complete (shorter) should go first (n=1) | - *The only thing I can think of for some reason, my brain prefers the first one because if this was given to me when I was feeling very unwell, say for example, like with the concentration thing, for some reason my brain likes the first one because it’s just a shorter question. Whereas, on the second scenario, the longer question is first, and my brain doesn’t seem to like that. Is that a fair comparison? (AM04 M4059_C)* |
|  | **Appropriate** Flow is better with activities first due to severity/specific nature of mobility (n=8) | - *“I guess you'd probably want to escalate the severity of the questions. So, by asking about getting around inside and outside before asking about day-to-day activities, that seems like a de-escalation of the severity of the question. ….The first question is sort of, if you have problems with getting around inside and outside, you're definitely going to have problems doing day-to-day activities. So, it almost seems as though your answer to the first one is likely to be as… from a mental point of view, it's kind of like you're asking a question about whether you feel do you feel good about yourself and you say do you feel suicidal? Whereas if you say, "Do you feel suicidal?" And then, "Do you feel good about yourself?" you've already asked a much more serious question before you ask the much more pleasant question. So, that's just the thought in my mind, that's all. I mean, but if somebody's reading the questions, I don't think it really matters. (AM06 M4059)* - *I think with the day-to-day activities it's more general so it makes you sort of think about your day and then the second one is more specific physically are you struggling to get around your house for example. So they seem to lead to each other better on the top one [note: activities were first on the top one] (AM07 F4059)* - *I prefer one because that seems a little bit more logical to me, I think day-to-day activities encompasses everything, are you getting around? I guess with question two, you're trying to be a bit more specific. Like I said, I don't think it's needed, but I'm not the researcher. So I think one first general, two, specific. (AM08 M60+)* - *“I think I would prefer the top one [note: activities were first on the top one] simply because I think question one kind of leads into question two rather than the other way around.” (AM09 M60+)* - *I think the first one you're talking about day-to-day activities and then the second question it's kind of broadening the aspect of the question it's then about going out… sorry that's question - the second half is one where you're talking about going outside. I suppose that that is a follow-on question from how you getting on kind of thing. (AM12 F60+)* - *"I think question, the question of getting around inside or outside is more of a follow-up to first question [...] I think day-to-day activities are also going around inside or outside for everyone. So, I probably say it's best having the getting around inside or outside as a second question cuz then it's more of like er, what's it called? Naturally flown interview" "getting around inside and outside is a bit for my attention span [...] I'll be like ugh it's really long I don't want to read it" "first one is doing day-to-day activities that's easy I'll fill it out but then I'll think okay I've filled the first one out might as well do the second then it'll be a snowballing effect I guess" (VG01 M1839)* - *"No, all I would say is I feel like getting around inside or outside is a day-to-day activity in a sense. But obviously you want more information on it specifically. So I think that first order makes more sense because of the fact that getting around inside or outside is like a category of doing day-to-day activities in my mind." (VG05 F1839)* - *Because they are related in a way but one is more specific than the other. So maybe the first one let's say the same thing first is more generic and the second one is getting a bit more into details of why you have problem with day-to-day activity" (VG09 F4059_C)* |
|  | **Appropriate** Activity item should go first because it’s more important (n=2) | - *"Yeah, I like the top one more. It seems more intuitive. Like you would put the day-to-day activities first. I think probably because the day-to-day activities is more essential to life. (VG04 M1839_C)* - *"I don't think it would affect how I filled it out at all, but I think that the top one makes slightly more sense intellectually…They're two different categories of thing because people can have mobility issues. They can be in a wheelchair and yet still not have problems with doing housework or shopping or whatever if it's a good wheelchair and they're used to using it. So in they're two different categories and I think the first one the day-to-day activities is actually somewhat more important because that's how it actually affects your functioning whereas getting around affects how you achieve your functioning if you see what I mean." (VG10 F60+)* |
| Prefer mobility first | **Appropriate** Mobility is part of activities, asking it first leads to considering different issues in the activity question because they have already answered about mobility (n=1) | - *"I guess so when I was talking about the day-to-day activities I was saying that like the way I’ve had to learn to think about activities is what are all the components going to be? And some of those components are how am I getting around? Erm and so I think for me it probably makes more sense to have getting around come first so you're dealing with that as a separate thing so and then you've already addressed that and then you can move on to thinking about in general day-to-day activities." "**if I saw the top one and I'd done the day-to-day activities, then I would have answered it partly thinking about getting around outside. And then if the next question was getting around inside/outside, then I'm like, " Oh I've already kind of factored that into my first question." Um whereas if it's the other way around, then I already know that that's what's happening" (VG2 F1839)* |
|  | **Appropriate** Mobility is part of activities, asking it first prompts considering other issues in the activity question, including activities outside the home (n=2) | - *I think I like the version that talks about the mobility first…. different from the one that I just answered, the new version…. Because I think, actually on reflection, day-to-day activities are all of the things that I might do in a day that do go beyond the house but that wasn't my first instinct. So, it gives, kind of, a gentle prompt to actually think, what do you do across the whole day and on a more average day, whereas, like I say, my first instinct was just to think of those things inside the home (AM02 M4059)* - *“it may be better the second way around [note: mobility first] because if you think about all these things, the problems that you have inside and outside… It may help you with what you do in your day-to-day activities, it might make you think in a more rounded way. (A10 F60+)* |
|  | **Appropriate** Flow is better with mobility first (n=4) | - *"I feel like if you ask about your mobility first, it would make more sense because that impacts your day-to-day activity" (VG06 F1839)* - *"I think more I look at it, the more I think the bottom one is the more sensible logical way of doing it because you're doing the walking. You're getting around first. That's the activity. You're getting out of your chair. You're getting out of bed, you're getting walking before you do the day activities. So yeah, even chronologically it makes sense."” (VG08 M60+)* - *"I think the second order is more logical. Sort of start off with the broader thing and then go down more into the specific thing. So, I think the second order is a bit more logical for me, but I don't think it would make any difference to how I would actually respond. " (VG13 F60+)* - *"If I was to if I was to set it out, I would have the second box, getting around followed by day-to-day activities. That's the way I'd set it out. Getting around is the more general one and it has the biggest impact and that affects the day-to-day activities. That's the most important one that affects the second one really." (VG14 M60+)* |
| No preference (n=6) | Interpretation would be the same (n=5) | - *“I really don't think it matters either way because they are so interconnected and overlapping. I wouldn't think that seems to be in the wrong order. I'd be absolutely fine with either of those because to think about your day to day things, you have to think about how you'd move around doing it and vice versa. So, no, to me I wouldn't feel any difference in the way that I've completed the survey if they were presented either way round” (AM01 M60+)* - *I don’t think answering them the other way around would make any difference to my overall responses for both. I don't think they influence each other for my view...no, no Preference whatsoever really. I don't, I see them both the same regardless. (AM03 F4059)* - *No, I don't think so. I think it's just… No, I don't think it really matters whether it's first or second. I don't think it would make your answers any different….No, I think they're both as relevant as each other, really. (AM06 M4059)* - *"But I think yeah, changing the order but doesn't really have I think for question for the day-to-day activities one I think yeah I don't interpret it any differently. I still interpret the same way." (VG03 M1839)* - *"And truthfully the answers that I've given you already are probably what I would give you again if that makes sense because I don't see any other options than what I've already talked about" (VG11 M60+)* |
|  | Preference may just be because that was the order seen first (n=1) | - *"I preferred it the way I saw it originally, but that might be because that's the way I saw it originally." (VG07 F60+_C)* |
| Unclear | (n=2) | - *I think I'd still give it the same score, but I think personally that the first one is more relevant to the second one. And I think you if you can't do day activities you probably won't be able to get outside. I think day-to-day activities are very very important like things like being able to get yourself ready etc. Not necessarily working, shopping or housework. Someone else can do that. ....because I think that that is the most important thing being able to do your everyday day-to-day activities (AM11 F60+)* - *Participant changed their mind a number of times (VG12 M60+)* |

## Table S8a: Summary of the themes for perceived difference between version of the item Get around: and vs or

| **Preference** | **Theme/justification** |
| --- | --- |
| They are different (n=21) | - One version (‘and’) has more clarity (n=1) **[Clarity]** - Difference based on literal interpretation of ‘or’ and ‘and’, with ‘and’ requiring problems in both domains (n=20) **[Alternate vs inclusive]** |
| They are the same (n=3) |  |
| Missing (n=3) |  |

## Table S8b: Themes and quotes supporting preferences for modification of the item Get around: and vs or

| **Preference** | **Theme** | **Quote** |
| --- | --- | --- |
| Prefer ‘or’ (n=14) | **Appropriate** 'Or' applies even when they have not been outside in last 7 days (n=3) | - *“probably “or” is better because some people are housebound so they would think, getting around outside would be nice but I can't.”* (AM01 M60+) - *"And so I think the first one [or] covers it better…So like if I'm someone who is housebound, then the first question I feel like that I could, I could consider it and have no issues about whether that applies to me or not.* *If I'm housebound and the question says inside and outside, I have a moment of thought of like but I don't go outside, so how does this apply to me?"* (VG02 F1839) - *“I think or covers both and I just feel like it flows off the tongue better getting around inside or outside whereas getting around inside and outside … So the reason why is because like for example I haven't been out this week so I've only in the last seven days been getting around inside not outside. So if someone did take that literally, they would read that and go, " but I haven't gotten around inside and outside. I've only gotten around inside. I wonder that matters, I wonder if I'm answering this correctly if I answer it." So I feel like or is a better use because you don't know if they've been outside." (VG05 F1839)* |
|  | **Appropriate** ‘Or’ is preferable when abilities vary considerably between two settings (n=10) | - *“I think the top one is more important to have “or” rather than “and” because some people can find that they can get around fine inside once they're in their own environment, but then outside is difficult with, uneven pavements or navigating maybe a steep incline. So I think having “or” is important there.”* (AM05 F4059_C) - *“you're asking you could have problems inside or outside or both. So, you can answer in a way that that's true for both of them. Whereas, if you're asking about do you have problems getting around inside and outside, people perhaps might conflate the two in the answer.” (AM06 M4059)* - *“If you're only going to ask one question, I prefer the first one because you're basically saying, are you mobile? And if you can only do inside, that's mobile. or because if you can get around inside, … if you get around outside, you can get around inside, I would guess, but maybe you can't.”* (AM08 M60+) - *“I think it's better putting or ...inside or outside because people may think it's a joint question so I maybe don't have so much problems because I don't have as many problems outside. So, I think having it separated with or is a better way to word it.” (AM10 F60+)* - *"I'm going to stick with or because some people may not have a problem indoors, but have a problem outdoors. And so therefore that makes it a more sensible word to use Or"* (VG08 M60+) - *“The first one's [or] correct, the second one's [and] wrong. It's inside or. For me it is." "I've got two different ways of getting around inside...I don't use, very rarely use a stick inside at home I'm walking about. Inside in a public building yes, I use a stick cuz one I've got it with me and I won't ever let go of the thing and usually they're bigger spaces. They're not spaces I'm used to."* (VG14 M60+) - *"I think I have a preference for the or one cuz the end one means maybe there might be a slight temptation unconsciously to select no difficulty for example if you're only having difficulty in one of the cases so whereas with the or one it's clear that either if you're having difficulty with either of the cases then it applies."* (VG03 M1839) - *“which one would I think it better is the ‘or’ … Because the ‘and’ is forcing you to have both of these things. And again, it's not like that" (VG06 F1839)* - "I would have a preference with ‘or’. … So it's very different to me because one is like it could be that you have a problem just getting around outside or if you have just problem getting out around outside then you will say you have difficulties … if I think of my daughter, she would say I have difficulty but both in the second one [and] you have to have difficulty in both cases and that will be say I have no difficulty because it's different. Some people might have a difficulty only outside and in this case you would say no difficult because you have to have both to answer". (VG09 F4059_C) - “The first one [or] is preferable" … “if you have a situation like the one I described where somebody can easily walk 10 or 20 feet but not further, then they can get around okay inside because inside there's furniture you can hang on to and chairs you can sit on and it's easy to arrange your furniture so you can sit down every 20 ft etc etc. But outside you can't do that. You can't rely on there being somewhere where you can sit every 20 ft. So they are really two separate problems, " (VG10 F60+) |
|  | **Appropriate** ‘Or’ is preferable because it’s a single question (n=1) | - *"If you were just keeping it as the one question, I think with the or rather than the and. I think with the and, like I say, it sort of says it's two questions to me whereas the ‘or’ is just the one single question." (VG12 M60+)* |
| Prefer ‘and’ (n=5) | **Appropriate** ‘And’ is more comprehensive (n=5) | - *“So it would be all of the activities within the last seven days. Whereas for the first one, maybe even subconsciously because you were right to, flag it to me, that I might have subconsciously just picked one or the other. …, I think the second one feels like you would get the whole view of an individual whereas I might have, without even knowing I was doing it to be honest, I might have just picked what I did inside the house” (AM02 M4059)* - *“I prefer the second one because I think it's much more to the point, and it's more encompassing. does that make sense? for me, it's more encompassing. when I think about my own condition, it makes me think about both of them for some reason. I don't know why, but I start to think about okay, how are things inside for me and how are things outside for me? Whereas the “or”... I start to almost question whether or not it was… I'm good inside and whether or not I'm good outside. I know that I start to question that myself about that. Whereas that makes me actually think explicitly about being in those situations.” (AM04 M4059_C)* - *it's getting you to think about them both whereas saying inside or…the way my mind works I'd perhaps think about one or the other rather than both of them. (AM09 M60+)* - *“the last one, because I think that it covers both doesn't it? says and rather than or.” (AM11 F60+)* - *“if I did have a preference, it's probably “and” so that they feel like its covering… when you're answering that question, it's going to cover both issues for them, inside and outside. They don't want to be having to clarify but only inside or outside… by answering the question when you ask “and”… when you say and you're covering both issues aren't you, inside and outside.” (AM12 F60+)* |
| Prefer two separate questions (n=2) | Prefer two separate questions (n=2) | - *“I would say that those questions need separating if you want a meaningful answer because interestingly my mobility is quite different inside to outside because inside it's flat and soft… outside you've got rugged pavements and if it's wet or slippy then that can be hugely different. So if I was to sort of answer it for both, I'd be giving a false answer to the outside by giving you one answer for the inside. See what I mean?” (AM03 F4059)* - *“But again, I'd probably like to see the question split anyway." (VG13 F60+)* |
| Preference depends on intention (n=1) | Depends upon whether you want to know if they have a problem with either or only if they have a problem with both settings (n=1) | - *"say you have difficulty outside and not inside. If it's ‘and’ you have to say no because it's not both. If it’s ‘or’ you have to say yes if it's either. So there are pros and cons either way aren't there." "You really need to be asked the two things separately. I'd say" (VG07 F60+_C)* |
| Preference based on misunderstanding (n=2) | Prefer ‘and' because it is less confusing because you have to have problems with both (n=1) | - *"The bottom one is less confusing. The top one I feel you'd have a lot of people filling it out as a lot of difficulty even if maybe they do have they are fine walking around indoors or something it's quite easy to misread it" (VG04 M1839_C)* |
|  | Prefer 'or' but because they incorrectly assumed invite to further detail (n=1) | - *"I think the first one will get you more information […] Could be that I'm having difficulties getting around inside the house, not outside and by you asking inside or outside, then I'm given a chance to say, actually, oh I'm having problems with this. But then if you say going around inside and outside, then it's just making them more general [...] doesn't really give me an invitation to say what my problem is" (VG01 M1839)* |
| No preference | n=1 | - *“I think they're both the same” (AM07 F4059)* |
| Unclear | n=1 |  |

## Table S9a: Summary of themes for perceived differences for versions of the item Concentrating/Thinking clearly: Difficulty vs Trouble

| **Preference** | **Theme/justification** |
| --- | --- |
| They are different (n=14) | - Difficulty is more severe (n=8) - Trouble is more severe (n=1) - Difficulty implies a problem from the start of a task or getting started (n=2) - Trouble implies the problem is due to external factors (n=1) - Difficulty implies an external or medical cause (n=1) (+2 secondary reason) - Difficulty is more objective (n=1) |
| They are the same (n=12) |  |

## Table S9b: Themes and quotes supporting preferences for modification of the item Concentrating/Thinking clearly: Difficulty vs Trouble

| **Preference** | **Theme** | **Quotes** |
| --- | --- | --- |
| Prefer ‘difficulty’ (n=17) | **Appropriate** More accurate (less ambiguous) and appropriate term to use for describing problems with concentration/thinking clearly (n=12) | - *“I would say difficulties a better word to use... difficulty is more of an expression of… it's causing you an issue. Trouble is not as narrow a definition when you think about the concentration side of things. Are you having trouble or having difficulty doing it? I think I'd say difficulty is more of an accurate word to use.” (A01 M60+)* - *“And in terms of which I think is kind of my preference or which I feel is better… I think “difficulty” feels more specific. to me it's enabling me to kind of concentrate more on the question and what I think has been looked for here. “trouble” feels a bit vague... that I might be wondering quite what's meant by trouble but difficulty to me feels precise and if I was to pick a preference it would be “difficulty” (AM02 M4059)* - *“I think it sounds too much almost like a euphemism… that does that… the word trouble compared to difficulty. Difficulty is a harder word that encapsulates maybe how someone might be struggling.” (AM04 M4059)* - *“I think I prefer difficulty actually, I'm going to say I prefer difficulty because that suggests that it's an ongoing problem rather than just something that's happened. Everyone can think I've probably just had trouble concentrating and thinking clearly about this question, but that's not something that's going to go on for the rest of the day.” (AM05 F4059)* - *“I think difficulty is a better term to use. It implies that it's problematic or it's causing problems. but that's probably just me. I think I just prefer the first version….It's how I would phrase the question if I was asking somebody.” (AM06 M4059)* - *“Trouble has different meanings and different connotations. you can be in trouble with the law because you've done something wrong, can't you? And it means that. But when you're talking about health, it means you're having difficulty with your health. You're having trouble with your health. It has the same meaning, but I think it's more direct when you ask somebody about their health. If you use the word difficulty than trouble.” (AM12 F60+)* - *“if you're talking about from the impact of physical problems and I would say difficulty.” (AM09 M60+)* - *“because it sort of explains, the amount of problem or trouble that you have. It can be quite difficult rather than trouble. I don't know. I don't know. I just reading it the difficulty sounds better. it sounds more explanatory.” (AM10 F60+)* - *“difficulty to me means you're struggling with it because of your health. so I think that is a good word to use. It's difficult for you… trouble could mean you can't concentrate because you're looking at something else. You're not concentrating on what you should be concentrating on. So, I think if you're talking about a health condition, difficulty is more… because you can't concentrate because it's difficult for you, not because you just can't concentrate because you're bored or something. You're bored with whatever you're watching or there's a difference. I definitely think regarding health conditions that difficulty is the best word to use.” (AM11 F60+)* - *"the top one is my preference because it has less ambiguity.* *Difficulty is how much problem you have with the concentration of thinking explicitly while trouble is a bit more ambiguous. It's because trouble is associated with getting into trouble as well as having trouble with something whereas difficulty is just having a problem with something" (VG04 M1839_C)* - *"I think difficulty is a stronger and more appropriate word in this context." (VG08 M60+)* - *"I think the difficulty it sort of the concentration levels dropping. Your fatigue setting in or something like that or you're just losing interest. I sometimes find that when I'm watching some of the programs on TV that my wife likes to watch. Whereas the having trouble concentrating is because you've got interruptions, somebody at the door, something happening outside." (VG12 M60+)* |
|  | **Appropriate** More aligned to the frequency response options (n=1) | - *"And then it'll confuse me when I'm answering later on. I'll say, well if it's a trouble, then why is it most all of the time? It's a difficulty instead." (VG01 M1839)* |
|  | **Appropriate** Trouble insinuates not able to concentrate or think clearly at all (n=1) | - *“I think trouble to me kind of insinuates that you can't concentrate or think clearly at all. Whereas difficulty means you are able to but it's not as easy as you would like it to be.” (VG05 F1839)* |
|  | **Appropriate** Difficulty is a broader concept covering process during tasks and getting started on tasks (n=1) | - *"I do feel like they’re intending to ask the same thing but in terms of how I would interpret it, maybe somehow, trouble seems to feel like it happens while I'm doing it and difficulty covers both started on something and in the process of doing it." "Yeah, the difference isn't that big" (VG03 M1839)* |
|  | **Ease of reading** Difficulty is easier to read (n=1) | - *"I prefer difficulty just from like an English reading point of view" (VG06 F1839)* |
|  | **Less distressing or unpleasant to be asked** Trouble considered patronising (n=1) | - *"trouble felt slightly patronizing compared to difficulty. It's a bit like did you struggle? That feels a bit patronizing. Whereas did you have difficulty sounds more objective to me.” (VG07 F60+_C)* |
| Prefer ‘trouble’ (n=4) | **Appropriate** Broader concept covering process during tasks and getting started on tasks(n=1) | - *“Yeah I think they mean something different. Having difficulty it's like it’s awkward to do it at all. Whereas having trouble is like you can concentrate on something but you have problems with it. Difficulty. It's sort implies that I struggle right from the beginning. It's just specifically for me like I can start and there's no difficulty in starting to concentrate on something but if that concentration is required over a period of time I have trouble with it then so I'd go for the trouble." (VG14 M60+)* |
|  | **Ease of reading** Simpler way of asking (n=3) | - *But I think trouble is a simpler, clearer way of asking because someone may think difficulty is a lot more severe than trouble because what are you looking for? Have you got anything? Yeah. Have you had any problems concentrating or thinking? and if someone thinks to themselves, it's not really difficult for me. I've had some issues, but I wouldn't say it's difficulty. whereby you're asking a much lower bar there, but then you're giving them the option how bad it is. (AM08 M60+)* - *"the two syllable word is better than the three syllable one, considering that some of the people doing this won't be native English speakers." (VG10 F60+)* - *"I think they mean the same thing but I think trouble is maybe a bit more accessible." "I think trouble’s maybe friendlier, maybe more accessible. " (VG13 F60+)* |
| Limited justification | Instinctive feeling (n=1) | - *"I prefer the second one [trouble] … Instinctual feeling" (VG09 F4059_C)* |
| No preference | No preference because they are asking something different (n=1) | - *I think they're just asking something different. So, I don't have a preference. I think both are relevant questions. but I think they are asking something slightly differently. (AM03 F4059)* |
|  | No preference because they are asking the same thing (n=3) | - *No, I don't mind either. (AM07 F4059)* - *"I can't see how I would read one as different from the other." (VG02 F1839)* - *"I've got to be honest with you, I'd say no preference really because that's how I feel about it really." (VG11 M60+)* |

## Table S10a: Summary of themes for perceived differences for item control: Lacked control vs no control

| **Preference** | **Theme/justification** |
| --- | --- |
| They are different (n=26) | - Lacked control is less severe or absolute than no control (n=25) **[Degree of control]** - Lacked control implies it is self-imposed potentially through lack of engagement (n=1) **[Lack is self-imposed]** |
| They are the same (n=0) |  |

## Table S10b Themes and quotes supporting preferences for modification of item control: Lacked control vs no control

| **Preference** | **Theme** | **Quote** |
| --- | --- | --- |
| Prefer ‘lacked control’ (n=18) | **Appropriate** Lack of control is broader in scope and includes less severe problems with control (n=16) | - *“… no control is quite some way further down the road of problematic than lacking control. So if I was driving a car and I was lacking control, I would feel I was still able to do that to some extent safely. Whereas if I had no control, I would be totally in the risk of crashing and I would apply that kind of scale in my life over these two differences and the other things.. with the examples.(AM02 M4059)* - *“I'd probably say the bottom one seems to be easier to understand, but I just prefer the wording of lacking control over something rather than having no control. cuz that seems like it's something that has gone on for quite some time and it can't be repaired. Whereas the top one, I think that you can lack control over something for maybe half an hour and then regain your control over it because you're thinking clearly or so. Yeah, I think I prefer lack control.“ (AM05 F4059_C)* - *It's a little bit more open ended the question because if you feel you lack lacked control, if you wanted to say I had no control, you could say most or all of the time. Whereas the bottom one as I say, it feels more as though it's a binary question. Did you have no control over your life? (AM06 M4059)* - *"No, no, I wouldn't answer them the same way. I think no control is very all or nothing. I think a lot of people feel like they lack control but to have no control is quite extreme. So yeah, that would be different for me." (AM07 F4059)* - *… “so I think lack control is a better way of putting it because you're not implying to someone that they have no control but they lack the control to do something but they could have done something if they really had the tools to do it” (AM08 M60+)* - *because to me, if I was reading “had no control” I would say I didn't have that at all because I have some control, but the lack control… it could be various extremes or… you know what I mean.. It just sounds like you could have variations of control or lack of control, but no control just seems too black and white. it's dead. you have no control. Nothing. (AM10 F60+)* - *“so lacked control is a little like you lack a little bit of control. that's how I see it. Whereas no control means it was totally out of your hands completely […]I think lacked is probably the best word because no control is… means that what happens to you is totally out of your control. I suppose it depends on the severity of the health condition that you're talking to someone about really because you can still have a health condition but have a certain element of control but some people have got no control i.e. people with dementia who someone else does everything for them.” (AM11 F60+)* - *"I think my preference would be lack control because I think if you say no control, you're going a lot of people who say none of the time because they still feel that they have some agency in their life. I would hope erm and then you're not c capturing any of the nuance of the bits that they don't have agency over or reduced choice" (VG02 F1839)* - *"Yeah, I say my preference is the top one because me personally I do have some level of control. I think everyone has some level of control. So it's maybe a bit more intuitive to that degree. It's the only way someone has no control over their life as if they're brain dead. You can still make the choice to say something at a certain time or everyone has at least a percent of a percent of control over their life. So I think lacked control is more intuitive in this regard " (VG04 M1839_C)* - *"I think lacked control suggests there was an element of control whereas had no control means no control and I think for many people it's not necessarily that they don't have no control but they feel that they lack it in that mentally they know what they need to do or what they'd like to do but can't always do that thing because physically they are restricted. That makes sense? Whereas I think no control suggests you can't do anything at all." "And I think people are more likely to lack control than have no control. But it really depends on the severity of their condition cuz there are actually going to be people that have no control. " (VG05 F1839)* - *"So, I feel like it should say lacked because for someone that could mean no control and for someone else that could just be minor like lack of control." (VG06 F1839)* - *"Top one for me personally lacked control...It indicates I had some control over something that happened today." (VG08 M60+)* - *"So for me it's a slightly different meaning...lack control will be that even if you lack a bit of control you will answer that most of the time instead no control it means that absolutely you can't decide anything so I don't know what you are asking for in this case for me that's a different meaning " "The first one [lack]." (VG09 F4059_C)* - *"I think they're quite a bit different to be honest. Lacking control sort of says that you have control over what you're doing to a degree whereas no control is you just no control whatsoever. So again with my condition I perhaps lack control because I can still do things and even if I'm in quite a bit of pain I still have that choice to go ahead and do something. Whereas if I have no control, it's a case of all the pain just stops me doing everything yeah." PROBE: So am I right in thinking that perhaps you'd have a preference for the lack control? RESPONSE: "Yeah" (VG12 M60+)* - *"**I think I've prefer lacked control because I might feel that I've not got control in certain areas over my day-to-day life, but I have in others. Whereas, if I got no control, then I think if I was answering with no control, I'd be more inclined to be on the lower end of the score because I would think, well I'm not completely out of control. Whereas if I was lacking control, I might be more of a middle score. But again, for me, it would be more my own limitations rather than somebody imposing things on me. But I think it would affect how I answered the question actually on that one." (VG13 F60+)* - *"Yeah, I think lack control is far better. No control is too extreme." (VG14 M60+)* |
|  | **Less distressing or unpleasant to be asked** Lacked control less emotionally distressing to answer (n=2) | - *“The first one, I would prefer. Yeah I think so. I think the second one…Yeah, I it can almost… if that question was administered to someone like me, I would feel like… I think it could be quite triggering almost but again that's just my temptations… no control it's quite powerful that lack control it doesn’t hit as hard.” (AM04 M4059_C)* - *“And the second one is quite upsetting. feeling like you've got no control. I think when you said that to me, how often do you feel like you've got no control? I'm like, swear word. That's quite hard to deal with. Whereas when you said feel like you lack control, that's like, you might feel like that sometimes. It's easier to cope with (AM07 F4059)* |
| Preferred no control (n=2) | **Appropriate** ‘Lacked’ implies own responsibility whereas ‘no control’ is due to circumstances beyond your control (n=1) | - *I think lacked control… I think again implies maybe a lack of interest or you're not very well focused whereas having no control to me as I said earlier… when I worked, it wasn't lacking it was just I simply didn't have the control cuz I was having to live my life in a way that I didn't want to. I think lacking control kind of implies more of a not engaging…if you see what I mean because for whatever reason you can't be bothered or things are too much for you whether it's a medical issue, physical issue” “Just because, as I say, I think lacked control it implies more of a kind of not caring if you like. Whereas having no control to me it's due to circumstances beyond your control if you like.” (AM09 M60+)* |
|  | **Appropriate** Lacked control is normal whereas no control is a problem (n=1) | - *"I would say probably no control […] cuz then lacking control is I think it's normal, but then no control is something that we can discuss” (VG01 M1839)* |
| Preference depends on intention (n=5) | Lacked control preferred if more granular responses are required (n=5) | - *“Lacked control to me would be.. you've got some control but not the amount of control that you might want. Whereas no control is completely nothing. You don't have any control. That's how I would read those....I would read lacked as.. it's not the level of control I'd want. No control is no control. Its an absolute.” “it depends what you're trying to elicit from people is if they feel absolutely nothing or there's a degree of control that has been lost or is it total? I think that's what you need to think about. Are you talking about black and white or a graduated scale?“ (AM01 M60+)* - *“So, the difference between lacking control over something and having no control is very significant. the no control is black or white, isn't it? It's like yes, I had no control or no, I didn't have no control. Whereas lack control for me can be a spectrum.” […] “Again I would say that it's a different question. So it would depend on what you were looking to measure. If you were looking to measure the degree of difficulty somebody has over in their life the top one might give you a better answer.” (AM03 F4059)* - *“if you want to gather as much information as you can then… and you want to understand from people who feel they've just lost a little bit compared to what they had yesterday, then lacked.” (AM12 F60+)* - *"so I don't think I any if that either of the questions are better or worse than the other but I just interpret them differently and I will answer them differently. So yeah I don't really have any particular preference as to which one." "I think the no control is pretty strong. Whereas if you lack control it could mean you have no control or you still have a little bit of control. It doesn't, lacking control is not the same thing as having no control at all." "if I see the question about lacking control I would be tempted to give maybe a higher answer than the no control one simply because of lacking control is a weaker statement." (VG03 M1839)* - *"Lacked means that there was some control missing whereas no control means absolutely no control. To me. If you lack control, you might still have some control, but I feel a lack isn't a complete lack necessarily." “But then if you're wondering about a slight feeling of not having control, lack would be better or reduced or incomplete or I don't know" (VG07 F60+_C)* |
| Did not like either (n=1) | Preferred alternative phrasing (n=1) | - *"I don't think that either of them is a terribly good way of phrasing it because unless you are incredibly rich and in complete health, nobody has complete control over what goes on in their life. I think a better phrase did not have as much control as you would like over your day life. " (VG10 F60+)* |
| Unclear (n=1) | Preference unclear although judged lacked control is less extreme | - *"Well when you've got no control, it suggests to me that it's completely out of your hands to a certain extent. Things just overrun you really. If you lack control, it's that you have still got some control over it, but not as much as you would want. It stops you from doing, shall we say, certain things, but you can still manage to do them, if that makes sense" (VG11 M60+)* |

## Table S11a: Summary of themes for perceived differences for modification explanation in brackets in control item modified version vs original version

| **Preference** | **Theme/justification** |
| --- | --- |
| They are different (n=2) | - The first simpler [**Simplicity] (n=1)** - Coverage is different and the first question better matches the explanation in the second [**Coverage**] (n=2, *one person provided two explanations)* |
| They are the same (n=1) |  |
| Missing (n=23) |  |

## Table S11b: Themes and quotes supporting preferences for modification of item control: explanation in brackets modified version vs original version

| **Preference** | **Theme** | **Quote** |
| --- | --- | --- |
| Prefer modified wording in brackets (n=2) | **Ease of reading** (n=2) | - *"I think the first bracket makes more sense to me. I think the second one is a bit of a tongue twister or a mind twister in that I don't know. It felt more complicated reading it where the first one just kind of flowed." (VG05 F1839)* - *Oh yeah, the top one. [laughs] Yeah ...They just want to keep it like, I have enough misery in my life, let alone on forms just being extra wordy and I don't know feel like you're trying to be tricked in forms to say something that answer a question how they want it to be answered. So yeah, top one for me on that.” (VG06 F1839)* |
| Prefer original wording in brackets (n=2) | **Appropriate** clearer/more accurate explanation (n=2) | - *"The second explanation. I think the second explanation is clearer. " (VG09 F4059_C)* - *"I think the second one's better because I think one of the things is when you have people coming in to help it can be quite difficult because they don't always ask how you would like smaller things done and that can make you feel out of control even if it's only a small thing and it can make me get a bit cross and a bit ratty. …. So I think that second one is good because it's got that bit in about how you like things and how you want things not necessarily how you need things done. " (VG13 F60+)* |
| Missing | (n=22) |  |

## Table S12a: Summary of perceived differences between response options ‘a little of the time’ vs ‘only occasionally

| **Preference** | **Theme/justification** |
| --- | --- |
| They are different (n=10) | - Only occasionally is less often (n=4) **[Only occasionally is less often]** - Only occasionally is more variably defined and could be interpreted differently by people (n=2) **[Only occasionally more variably defined]** - Only occasionally is sporadic may not occur every day (n=2) **[Only occasionally is sporadic]** - **[Only occasionally is more specific] (n=1)** - **[Only occasionally not distinct from sometimes]** (n=1) |
| They are the same (n=14) |  |
| Missing (n=2) |  |

## Table S12b: Themes and quotes supporting preferences for modification for response options ‘a little of the time’ vs ‘only occasionally

| **Preference** | **Theme/justification** | **Quote** |
| --- | --- | --- |
| Prefer ‘a little of the time’ (n=16) | **Appropriate** More suited to the scale when thinking about last 7 days or events that are not rare (n=2) | - *“I like “a little of the time”.* *I think that that fits the scale to me better than occasionally.” (AM02 M4059)* - *“very occasionally feels like it shouldn't be there in the last seven days. Very occasionally feels like a once in a year, once in a long while. But a little of the time when you're talking about that specific seven days, it does feel like that fits within that time frame better.” (AM12 F60+)* |
|  | **Appropriate** A little of the time less ambiguous (n=4) | - *“I would say “a little of the time”. Because as I say, to me, “only occasionally” it's a bit less defined than “a little of the time”. (AM01 M60+)* - *“I think the first one to think of things in amounts of time is easier and less abstract than words like occasionally....” (AM07 F4059)* - *“I just think it's simpler and it's not… what's the word**? It's less ambiguous” (AM10 F60+)* - *"I think a little of the time makes more sense. " (vG05 F1839)* |
|  | **Appropriate** A little of the time distinct from sometimes while only occasionally is not (n=3) | - *"I think that strong preference for a little.* *There's a difference between little and sometimes. There isn't really a difference between occasionally and sometimes." (VG03 M1839)* - *“"I think I prefer a little of - I don't know! …"I think it just flows better for me from none of the time to a little of the time. I think it's more defining for me like where I would put myself in that category than none of the time, only occasionally, sometimes. I'd be like is it only occasionally or is it sometimes? " (VG06 F1839)* - *"I think if I only had occasionally I might go into sometimes more and if I had a choice of a little of the time I might choose that one. So I think I prefer a little of the time." (VG11 F60+)* |
|  | **Appropriate** A little of the time is more consistent with the other response options (n=2) | - *I think a little of the time is more consistent with the other options…because it feels more consistent to me.” (AM06 M4059)* - *"I prefer a little of the time just because it's next to none of the time which and then most of all the time so it makes it feel a bit more consistent but I could go either way. It doesn't really matter." (VG04 M1839)* |
|  | **Less distressing or unpleasant to be asked** Only occasionally minimises the issues (n=2) | - *“I would ask why the word only occasionally is in there. it kind of minimizes some things there. I felt a little bit hmm not quite like the only because if you're feeling those things occasionally they are significant whereas the word only occasionally it is almost it feels minim though it's minimizing something. a little of the time doesn't carry any offense. not that only occasionally does carry some offense, but it's just that word only.” (AM03 F4059)* - *"The word only always sounds like it has some value judgment in it […] that would be my only objection to it of just like if something is only something, you've already built in some assumptions there I feel. I like a little of the time." (VG02 F1839)* |
|  | **Ease of reading** A little of the time is simpler (n=3) | - *“I think it just makes the form a bit simpler. I just think that they are the same occasionally and a little of the time, but I think some people might find it difficult to clarify what occasionally means, whereas I think it's very simple to say a little of the time “ (AM05 F4059)* - *“it just seems more straightforward…..” (AM08 M60+)* - *"A little of the time I think …"A little of the time is better for the same reasons as before, only occasionally might stump somebody who's not a native English speaker. (VG10 F60+)* |
| Prefer ‘only occasionally’ (n=7) | **Appropriate** Easier to understand than a little of the time (n=5) | - *“So I think that is a much more specific… pins you down a bit more than a little of the time, which I think it's a bit woollier...just because it's more specific to me.” (AM09 M60+)* - *“I just think it's more simple and I sort of understand it more than a little of the time” (AM11 F60+)* - *"I prefer only occasionally. It's clearer to me…they mean the same, but it seems clearer for me to say only occasionally, it’s more, yeah they mean the same but for me it’s clearer compared to a little of the time, it sounds weird a little of the time for me, yeah" (VG09 F4059_C)* - *"I would prefer I think the only occasionally one on that one. Yeah. Again, I'm struggling to explain why, but it just seems a more appropriate one. So taking the feeling exhausted one. I would struggle to determine what a little of the time is. Whereas occasionally I can say well yeah every second day in the afternoon at 2 o’clock I feel exhausted and that is only occasionally. A little of the time is well, when is that? Why? Is it a lot of the time relative to the day or a lot of the time relative to what I'm doing at that particular moment in time. " (VG12 M60+)* - *". I do like only occasionally because you never get that. It could be on something that it may happen one day but not the next day …It [only occasionally] just seems a bit more specific than a little of the time." (VG14 M60+)* |
|  | **Appropriate** Only occasional fits the scale better (n=1) | - *"I prefer only occasionally because some things are only occasional and you can't put none because they're only occasional. But you don't want to put sometimes because it implies it's more often. And a little of the time makes it sound more regular." (VG07 F60+)* |
|  | **Ease of reading** Only occasionally is more user friendly and/or clearer (n=1) | - *“I do prefer only occasionally for some reason because I think in terms of the questionnaire, the questionnaire is easier to interpret but just looking at it, when I look at the two I can see the question easier and from a semantics perspective I don't think there's any significant difference but from an interpretation, from a usability perspective, only occasionally from my perspective it is more user friendly.” (AM04 M4059)* |
| No preference or no clear justification | Only occasionally preferred but no clear justification given (n=3) | - *“I don't see to be honest people saying only occasionally […] I think it makes more sense to have only occasionally I just don't know why I purely it's just a mind thing that I like the way that it looks. That's it" (VG01 M1839)* - *"And then I would put occasionally, sometimes often. I would drop the word only altogether." (VG08 M60+)* - *"Probably no preference really, yeah no preference to be honest." (VG11 M60+)* |

## Table S13a: Summary of perceived differences between response options ‘moderate’ vs ‘some’

| **Preference** | **Theme/justification** |
| --- | --- |
| They are different (n=19) | - Moderate is more severe (n=13) - Moderate is less severe (n=1) - Moderate is a different measure (n=4) - Some is too specific (n=1) |
| They are the same (n=7) |  |

## Table S13b: Summary of themes and quotes on preferences for response options ‘moderate’ vs ‘some’

| **Preference** | **Theme/justification** | **Quotes** |
| --- | --- | --- |
| Prefer “moderate” (n=15) | **Appropriate** Better given scale (n=10) | - *“Some” to me is more of a quantity expression and “moderate” is more of a quality expression. So I'm thinking the severity would be moderate but “some” would be… do we get it on a scale of a lot… not time wise…I would say personally moderate, but some might be more inclusive for other people.” (AM01 M60+)* - *“I think it's more precise for me just in terms of capturing how I would feel because some I don't think would be moderate on the scale. Maybe it's what I'm used to” (AM04 M4059)* - *“I prefer moderate actually because slight difficulties is some difficulty, isn't it? So moderate to me is different to slight whereas some isn't different to slight in my view.” (AM03 F4059)* - *“I think moderate makes it different saying it's more than slight. Whereby some and slight… it's asking the same thing or it's the same answer to that is… I would say some difficulty is slight difficulty.” (AM08 M60+)* - *“I would say moderate is the best word because moderate mean… slight is you just have a little bit and moderate means it's a little bit, in the middle. It's not just a tiny bit. And some difficulty to me is not that much different to slight” (AM11 F60+)* - *“I like moderate. But I don't know. No, I wouldn't say one is better than the other. It's kind of asking the same thing, but it can be interpreted slightly differently…..I think it's kind of describing a level of pain, isn't it? it's a level of whatever. it's not quite bad and it's not, very mild. it's kind of in the middle. Whereas some could be, 3 weeks ago and next time in six months, that is some. it's not giving you.. it's not describing a level, is it?” (AM12 F60+)* - *"I think the transition from slight to moderate and then from moderate to a lot of difficulty [is more] makes more sense than slight to some and then to a lot of difficulty cuz that just jumps through one option" … "the first one is my preferred one erm because the gap from slight to moderate feels like the same gap from moderate to a lot of difficulty whereas slight to some feels so small to jump then from some to a lot of difficulty is leaving this blank gap where I might feel I'm in that blank"" (VG01 M1839)* - *"so I think moderate makes more sense as a step up erm I feel like most people know what moderate means" (VG02 F1839)* - *"I guess for me it seems like the difference between slight and moderate is bigger than the difference between slight and some. …I think I have a preference for the moderate" (VG03 M1839)* - *"So maybe it's more moderate because slightly and some might be the same. So moderate it makes it clearer that it’s more than some. Yeah. So moderate actually" (VG09 F4059_C)* |
|  | **Appropriate** Easier to understand (n= 4) | - *"sometimes when I'm being asked questions, just the more simplified the question is, the easier it is to answer it. So moderate is it's just a basic word" ((VG05 F1839)* - *"like moderate's on a scale whereas some is like an unknown …So moderate I would put in bang in the middle as like moderate when you think about things being moderate, like temperatures blah blah blah. Some, everyone can interpret that differently whereas I think moderate has a more solid meaning to people" (VG06 F1839)* - *"Moderate seems more in the middle between slight and a lot to me personally. But some is too vague really. It could be a little it could be lot. So, you're just judging it by its position in the scale instead of by the words in it. And I think it's important to get the word to reflect the intended meaning. " (VG07 F60+)* - *"I think I get rid of some difficulty. I think moderate difficulty just makes more sense" (VG14 M60+)* |
|  | **Appropriate** Reflects on-going difficulties (n=1) | - *"Again I think the top one [moderate difficulty] would be a better one. …I think moderate difficulty to me sort of implies a more ongoing thing whereas some difficulty might just be an intermittent type thing. So again with my walking my moderate difficulty would be I constantly have to keep stopping whereas some difficulty might be yeah I'm okay I can walk but I stop every now and then so not as descriptive" (VG12 M60+)* |
| Prefer “some” (n=7) | **Appropriate** Better description of difficulty (n=1) | - *“I think I like “some difficulty”. yeah to me that would be the one I would choose. “some difficulty” because I think it's more reflective of.. that there's difficult that could be happening some of the time.” (AM02 M4059)* |
|  | **Appropriate** Easier to understand (n=4) | - *“I think some difficulty is more simplistic. So I think some people might understand moderate to mean severe difficulty. So some difficulty I think the wording of this is much simpler and I'd say it's more user friendly really...**I think some people might find it difficult to categorize what moderation or moderate difficulty meant.” (AM05 F4059)* - *“I have a degree in English. So for me, moderate difficulty is fine. I know what that means. But if I was doing this with a child, they probably wouldn't know what moderate difficulty means. Whereas they'd know some” (AM07 F4059)* - *“I think saying some difficulties is perhaps a little more specific because for me moderates… I think some difficulty just narrows it a little bit whereas I moderate makes it a bit kind of more open to interpretation....if you look at the two either side as well, I think it fits better between the two” (AM09 M60+)* - *“I think some difficulty is more simplified for people” (AM10 F60+)* |
|  | **Appropriate** Better given the scale (n=2) | - *“I think it's just a subjective feeling... it just feels like a better progression. Moderate doesn't seem… moderate and slight seem... I'm not sure I can tell too much difference between them whereas slight and some feels more impactful. But again, it's just subjective.” (AM06 M4059)* - *"I think some is possibly, personally, I think is the better word. Slightly, slightly the better word….No slight some a lot makes sense in a logical progression of the slight, some whatever. No slight, moderate a lot…. I think no slight some a lot gives more gradual movement through the graduation. " (VG08 M60+)* |
| No preference or clear justification | Prefers “moderate” (n=3) | - *"But I would say moderate difficulty is probably more intuitive than some cuz some is a bit more variable. …Yeah, the top one would be my preference though I don't think it really matters" (VG04 M1839)* - *"Me moderate difficulty, but I have an extremely good vocabulary." (VG10 F60+)* - *"I think moderate difficulty just fits better " (VG13 F60+)* |
| Missing | (n=1) | Participant misunderstood the question. |
